# Engineering a spleen-selective mRNA-LNPs vaccine by decoupling the inflammation from cellular immunity-mediated cancer immunotherapy

**DOI:** 10.7150/thno.118976

**Published:** 2025-08-30

**Authors:** Xiaoke Gao, Meng Zhang, Shiyang Du, Lixia Ma, Xiaohan Yao, Boao Xie, Jiajia Wan, Yuqiao Sheng, Bo Qin, Wenjing Deng, Ningjing Lei, Wentao Mo, Ming Wang, Zhijun Sun, Zhihai Qin, Fazhan Wang

**Affiliations:** 1 Medical Research Center, The First Affiliated Hospital of Zhengzhou University, Zhengzhou University, Zhengzhou, 450052 Henan, China.; 2 Department of Neuro-Intensive Care Unit, The First Affiliated Hospital of Zhengzhou University, Zhengzhou, 450052 Henan, China.; 3 Department of Vascular Surgery, Peking Union Medical College Hospital, 100010 Beijing, China.; 4 Translational Medical Center, The First Affiliated Hospital of Zhengzhou University, Zhengzhou University, Zhengzhou, 450052 Henan, China.; 5 Department of Microbiology and Immunology, School of Basic Medical Sciences, Zhengzhou University, Zhengzhou, 450001 Henan, China.; 6 State Key Laboratory of Oral & Maxillofacial Reconstruction and Regeneration, Key Laboratory of Oral Biomedicine Ministry of Education, Hubei Key Laboratory of Stomatology, School & Hospital of Stomatology, Wuhan University, Wuhan, 430079 Hubei, China.; 7 Frontier Science Center for Immunology and Metabolism, Taikang Center for Life and Medical Sciences, Wuhan University, Wuhan, 430071 Hubei, China.

**Keywords:** mRNA vaccine, lipid nanoparticles, spleen, cellular immune responses, cancer immunotherapy

## Abstract

**Rationale:** mRNA vaccine-based cancer immunotherapy requires innate immune activation followed by potent cellular immunity. Vectors of lipid nanoparticles (LNPs) with proinflammatory properties activate the innate immune pathway, while excessive inflammatory response of mRNA-LNPs vaccine often results in systemic inflammation, compromising its therapeutic safety.

**Methods:** Here, we engineered a spleen-selective mRNA-LNPs (mRNA-sLNPs) vaccine by decoupling the excessive inflammation from strong cellular immunity through ionizable lipids substituting for potent cancer immunotherapy.

**Results:** The mRNA-sLNPs vaccine with reduced inflammation achieved superior mRNA translation in the spleen and enhanced antigen-specific cellular immune responses. Mechanistically, the optimized mRNA-sLNPs vaccine amplified lysosomal escape and boosted antigen presentation with moderate co-stimulatory molecule expression by mitigating TLR4/MyD88/NF-κB signaling and pro-inflammatory cytokine secretion. In therapeutic mouse models, the engineered mRNA vaccine significantly inhibited both the growth of subcutaneous B16F10-OVA melanomas and the development of lung metastases following intravenous injection of B16F10-OVA cells with augmented infiltration of CD4^+^ and CD8^+^ T cells in the tumor microenvironment.

**Conclusion:** Our findings might redefine the design principles of mRNA-LNPs vaccine as diminishing the inflammation of LNPs does not compromise cellular immunity, offering a clinically translatable strategy to advance mRNA vaccines for cancer immunotherapy.

## Introduction

Messenger RNA (mRNA) vaccines have achieved groundbreaking progress in addressing infectious disease challenges 1-3. Enabled by breakthroughs in neoantigen screening, delivery vectors, and adjuvant technologies, mRNA vaccines enable rapid design and personalization, demonstrating their transformative potential in clinical oncology 4-8. Timely immune activation is critical to curb the exponential growth of tumor cells and forestall the establishment of an immunosuppressive tumor microenvironment 9,10. The spleen emerges as an ideal location to initiate vaccine- induced immune response since its vascularized structure and high antigen-presenting cells (APCs) density facilitate rapid antigen processing and adaptive responses 11,12. However, precision delivery vectors are required to selectively deliver antigen-coded mRNA to the spleen to realize this potential.

Lipid nanoparticles (LNPs) represent the most advanced delivery vectors for mRNA vaccines 13, exemplified by their pivotal role in COVID-19 2,14,15 and respiratory syncytial virus vaccines 3. However, 80-90% of mRNA vaccine recipients experience adverse effects, with mild-to-moderate symptoms (e.g., pain, fever) linked to inflammation, while moderate-to-severe reactions occur in approximately 10% of cases 16. It has been reported that the components of LNPs, particularly ionizable lipids, play an essential role in activating inflammatory pathways and inducing inflammatory cytokines 17-21. These inflammatory adverse effects constrain dose escalation and broader biomedical applications of LNPs-based nanomedicines 19,22. Spleen-selective LNPs-based mRNA (mRNA-sLNPs) vaccines, as demonstrated by us and others 23-27, show potent anti-tumor responses through activating splenic dendritic cells by the proinflammatory adjuvanticity of LNPs. The encapsulation of mRNA by sLNPs also relies on ionizable lipids, inheriting the inherent inflammatory risks of LNPs, which may undermine both safety and translational efficacy of sLNPs 23.

Balancing innate immune activation and inflammation-mediated toxicity remains a critical challenge for mRNA-sLNPs vaccines 28. The induction of antigen-specific toxic T cell immune responses by mRNA-sLNPs requires APCs expressing antigen proteins and presenting them to T cells via Peptide-MHC Complex (pMHC) effectively as well as the activation of APCs for subsequent T cell activation, proliferation and differentiation 29. The pro-inflammatory properties of mRNA vaccines are necessary for antigen-specific toxic T cell responses, as antigen presentation in a non-inflammatory condition induces regulatory T cells (Tregs)-mediated tolerogenic responses 30,31. However, the over-inflammatory activity of mRNA LNPs might restrict the translation of antigen-coded mRNA and subsequent antigen presentation, resulting in inadequate anti-tumor cellular responses 18,32. It has been reported that fine-tuning the chemical architecture and stoichiometry of ionizable lipids within mRNA-LNPs enables precise modulation of their protein expression and inflammatory profiles 17,32. This chemical optimization paradigm shifts from conventional inflammation-enhancing adjuvants toward molecularly engineered immunomodulation might also preserve the potency of the vaccine while circumventing its systemic toxicity.

**Scheme 1:**
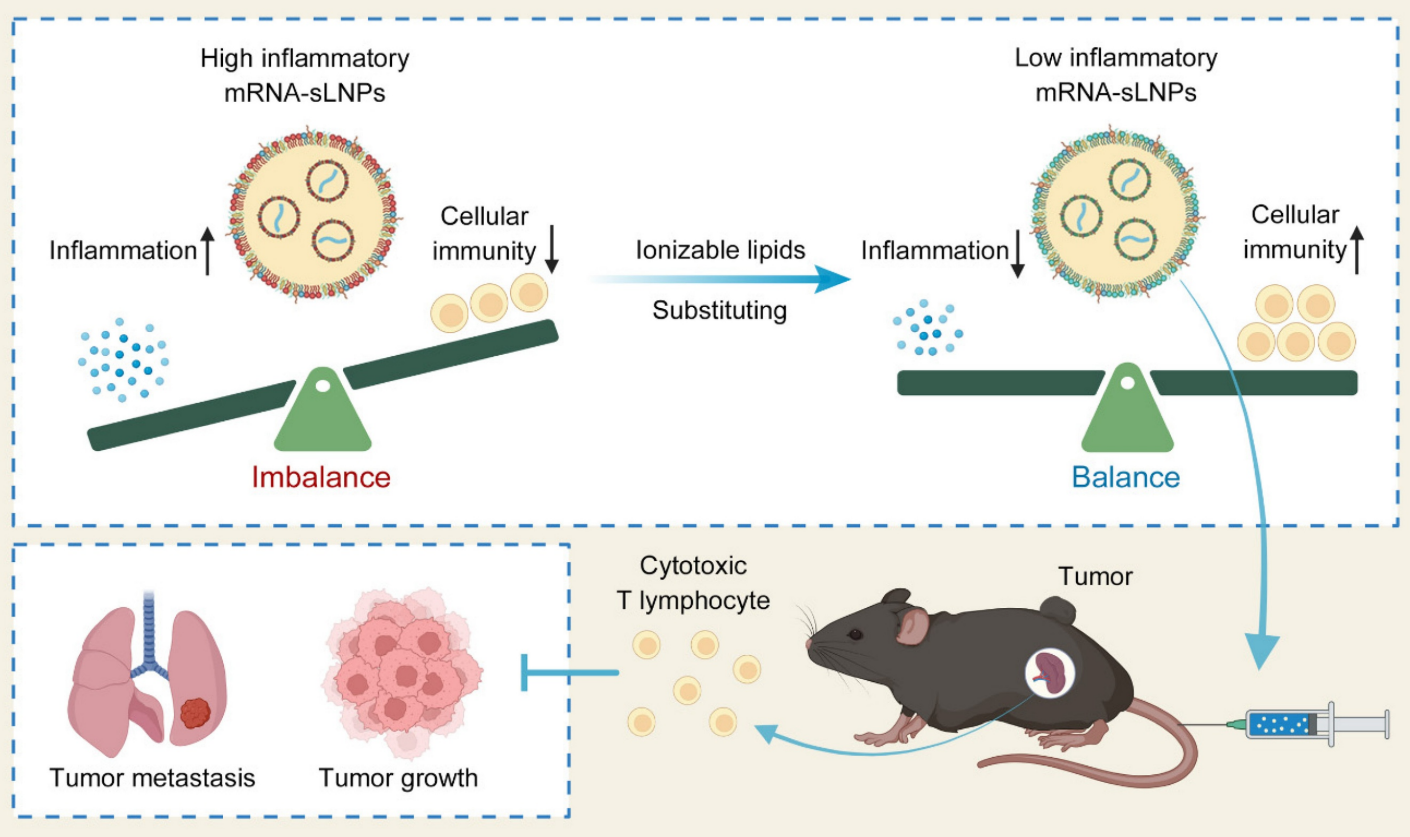
Schematic illustration of mRNA-sLNPs-mediated balance of inflammation and cellular immunity for cancer immunotherapy. The synergism of innate immune activation and potent cellular immunity by LNPs-based mRNA vaccine through ionizable lipids substituting for potent cancer immunotherapy (Created in https://BioRender.com).

Previously, we developed spleen-targeted mRNA-LNPs vaccines leveraging fatty acid metabolic pathways for potent and rapid antitumor responses, yet with restricted efficacy at low dosage 23. In this study, we fabricate a spleen-targeted mRNA-LNPs vaccine with enhanced mRNA delivery efficacy and reduced proinflammatory properties for potent antigen-specific immune responses against tumors by comparing two FDA-approved ionizable phospho-lipids of SM-102 and DLin-MC3-DMA (MC3). The physicochemical properties, mRNA delivery efficacy, proinflammatory properties, induction of antigen-specific immune responses, cancer immunotherapy efficacy, and preliminary biocompatibility of each formulation were investigated to optimize a potent mRNA vaccine with excellent clinical translation promise. By decoupling hyperinflammatory properties of LNPs from potent immunogenic cellular responses, our redesigned spleen-selective SM102-based LNPs enhance the translation of mRNA and tumor-specific toxic T cell responses without provoking deleterious cytokine storms. Unlike conventional inflammation-enhancing strategies 33-35, this novel inflammationdiminishing LNPs design paradigm underscores the synergism of innate immune activation, mRNA translation, and potent cellular immunity for cancer immunotherapy, providing a transformative framework to facilitate LNPs-based mRNA nanomedicines design and its broader biomedical applications (Scheme 1).

## Materials and Methods

### Materials

MC3, SM-102, DSPC, DMG-PEG (2000), and cholesterol were procured from AVT (Shanghai) Pharmaceutical Tech Co., Ltd. Stearic acid (SA) was obtained from Sigma-Aldrich (St. Louis, MO, USA). E. coli Poly(A) Polymerase, Cap 1 Capping System, N1-Me-Pseudo UTP, and T7 High Yield RNA Transcription Kit were obtained from Novoprotein (Shanghai, China). Cy5-UTp was purchased from Jiangsu Synthgene Biotechnology Co., Ltd. Phosphate buffer (PBS), citrate buffer, TAE buffer and Hoechst 33342 Stain solution were procured from Beijing Solarbio Science & Technology Co, Ltd. RNA Loading buffer (Denatured) and Gel-Red were purchased from Beyotime (Shanghai, China). GM-CSF, 2-Mercaptoethanol, DMEM, and 0.25% Trypsin-EDTA were procured from Thermo Fisher Scientific (Waltham, MA, USA). Penicillin-Streptomycin Solution and RPMI-1640 were obtained from Cytiva (Marlborough, MA, USA). Fetal bovine serum (FBS) was obtained from Invigentech (Irvine, CA, USA). D-Luciferin (potassium salt) was acquired from CSNpharm (Chicago, IL, USA). Table S1 provides a complete list of flow cytometry antibodies, while Table S2 contains full sequences of all qRT-PCR primers.

### Cell lines

DC2.4 and B16F10 cells were both purchased from the ATCC. DC2.4 cells were cultured in DMEM containing 100 U/mL penicillin, 100 μg/mL Streptomycin and 10% FBS. B16F10-OVA cells were cultured in RPMI-1640 supplemented with 100 U/mL penicillin, 100 μg/mL Streptomycin and 10% FBS.

### Mice

Female C57BL/6J mice aged 6-8 weeks were procured from SiPeiFu (Beijing, China). All animal experiments were ethically supervised and approved by the First Affiliated Hospital of Zhengzhou University Animal Care and Use Committee (2021-KY-0634-001).

### Synthesis of mRNA and mRNA-Cy5 *in vitro*

According to the kit instructions provided by the manufacturer, Nucleoside-modified Luc mRNA and OVA mRNA were produced using T7 RNA polymerase by replacing UTP with N1-Me-Pseudo UTP or Cy5-UTp. All mRNA was stored frozen at -80°C.

### Preparation and characterization of lipid nanoparticles

The lipid components (MC3/SM-102, cholesterol, DSPC, DMG-PEG 2000, SA) were formulated in ethanol, whereas the mRNA was prepared in citrate buffer (0.1 M, pH 4.5). Nanoparticle self-assembly was achieved through rapid mixing of the organic and aqueous phases at a 3:1 volumetric ratio. The primary lipid composition of the mRNA-sLNPs was formulated with SM-102 or MC3, DSPC, cholesterol, DMG-PEG and SA at molar percentages of 21.5:4.3:16.5:0.7:57. The mRNA-sLNPs were subsequently purified via ultrafiltration-mediated buffer exchange, wherein ethanol and citrate buffer were replaced with PBS. The PDI and zeta potential of mRNA-sLNPs were quantified by a Zetasizer Nano ZS90 (Malvern Instruments, located in Malvern, UK). Measurements were performed in triplicate with independent nanoparticle batches to ensure data reproducibility. Finally, the ultrastructural morphology of mRNA-sLNPs was visualized by TEM. For sample preparation, a drop of the mRNA-sLNPs suspension was deposited onto copper grids, followed by negative staining with phosphotungstic acid to enhance electron contrast. Encapsulation efficiency (EE) of mRNA-sLNPs was quantified by complementary orthogonal assays. First, gel electrophoresis was used for visual confirmation. Free mRNA (0.2 μg) or mRNA-sLNPs (equivalent mRNA dose) were denatured in 2× RNA Loading Buffer at 65℃ for 10 min and electrophoresed on 0.9% formaldehyde-agarose gels (supplemented with GelRed). mRNA sizes were determined by millennium™ RNA markers. Next, fluorescence-based quantification was performed with the RiboGreen RNA quantitative detection reagent kit (Beijing Solarbio Science & Technology Co, Ltd., Beijing, China). 2.4 μL of each formulation was diluted in 500 μL TE buffer and split: one aliquot was lysed with 2% Triton X-100 to expose total mRNA, the other remained intact to quantify free mRNA. After 10 min at 37 °C, samples were mixed 1: 1 with RiboGreen reagent (1: 200 in TE) and fluorescence measured (λex 500 nm, λem 525 nm, SpectraMax i3x, Lagerhausstrasse, Austria). EE was calculated as *[(Ftotal - Ffree)/Ftotal] × 100 %* against an RNA standard curve (0-1000 ng/mL). The gel imaging was performed with a Bio-Rad ChemiDoc MP system (Hercules, CA, USA). Additionally, the particle size, encapsulation efficiency and copy number were also characterized using the NanoFCM instrument (NanoFCM Inc., Xiamen, China) following manufacturer-specified protocols. The copy number was calculated as *the fluorescence intensity of mRNA-encapsulating LNPs divided by the fluorescence intensity of a single mRNA molecule*.

### Bioluminescence imaging and *in vivo* pro-inflammatory properties of MC3- and SM-102 sLNPs

*In vivo* bioluminescence assessment of murine organs was executed via an IVIS® Spectrum imaging system. Mice intravenously received either MC3- or SM-102-formulated sLNPs encapsulating luciferase-encoding mRNA (MC3 sLNPs-Luc or SM-102 sLNPs-Luc) (0.2 mg mRNA/kg) via tail vein injection. Bioluminescence imaging was preceded by intraperitoneal administration of D-luciferin substrate (150 mg/kg) 10 minutes prior to signal acquisition. Following euthanasia, ex vivo bioluminescence of excised organs (heart, liver, spleen, lungs, kidneys) was quantified to assess Luc-mRNA translation.

To assess the *in vivo* pro-inflammatory properties of sLNPs, mice were intravenously administered PBS, MC3- or SM-102-formulated sLNPs encapsulating ovalbumin-encoding mRNA (MC3 sLNPs-OVA or SM-102 sLNPs-OVA). Serum was collected 24 hours post-injection for quantification of IL-1β and IL-6 levels using ELISA kits (RUIFAN, Shanghai, China) per manufacturer protocols, with absorbance measured at 450 nm. For DC activation analysis, spleens were harvested at 24 hours, processed into single-cell suspensions, and stained with fluorescently labeled antibodies against CD11c, CD80, CD86, MHC-I (H-2K^b^), and MHC-I bound to SIINFEKL. Dead cells were excluded by DAPI staining. Samples were analyzed on a BD flow cytometer.

### *In vitro* cellular uptake and lysosomal escape of mRNA sLNPs in DC2.4 cells

DC2.4 cells were seeded at 5×10^4^ cells/well in 35-mm glass- bottom dishes and allowed to adhere overnight. The cells were subsequently treated with Cy5-mRNA encapsulated in either MC3- or SM-102-formulated sLNPs (MC3 or SM102 sLNPs-Cy5). Cells were incubated for 1 h, 3 h and 5 h, then washed with PBS. Then, the cells were incubated with Lyso-Tracker Green staining solution (GLPBIO, Montclair, CA, USA) at a concentration of 100 nM at 37°C in the dark for 1.5 h. Finally, the cells were washed with PBS, counterstained with 10 ng/ml Hoechst 33342, and then observed with a confocal laser-scanning microscope (Nikon, Tokyo, Japan). Images for all treatment groups were acquired using identical imaging parameters (e.g., exposure time, gain, laser power) to ensure signal intensity differences reflect biological variation. For flow cytometry-based evaluation of mRNA delivery efficiency, DC2.4 cells were seeded in 24-well plates at 5×10⁴ cells/well and cultured to 80% confluence. The cells were treated with MC3 or SM-102 sLNPs-Cy5 for 1, 3, or 5 h. Post-incubation, unbound nanoparticles were removed by three washes with PBS. Cells were detached using 0.25% EDTA-trypsin, washed twice in PBS, and resuspended in FACS buffer (PBS + 2% FBS). Cy5 fluorescence was detected by flow cytometry, with MFI analyzed via FlowJo and visualized using GraphPad Prism (V8; La Jolla, CA, USA).

### Generation of BMDCs

Bone marrow-derived cells were harvested from femurs and tibias of female C57BL/6J mice via cold PBS perfusion. Following centrifugation (4℃, 300 ×g, 5 min), cells were maintained in complete RPMI-1640 medium containing 20 ng/mL GM-CSF, 100 μg/mL streptomycin, 100 U/mL penicillin, 55 μM β-mercaptoethanol, and 10% FBS. On day 3 of culture, fresh complete medium was supplemented at 1:1 volume. After 7 days of differentiation, non-adherent cells were collected for downstream applications.

### Antigen presentation and activation of BMDCs by sLNP-OVA

To assess the antigen presentation and activation status of BMDCs by sLNPs-OVA, BMDCs were treated with PBS, MC3 sLNPs-OVA, SM-102 sLNPs-OVA or LPS for 24 h. Following incubation, the cells were harvested and incubated in FACS buffer (PBS with 2% FBS). The cells were labeled with antibodies of CD11c, H-2Kb bound to SIINFEKL, CD86, CD80, and MHC-II for 30 min at 4℃. After two washes with FACS buffer, cells were resuspended in FACS buffer and analyzed on a BD flow cytometer. Dead cells were excluded by DAPI staining. The experiment included three independent replicates with duplicate samples. Data were processed using FlowJo software (V10). The mRNA transcription of IL-1β and IL-6 in the BMDCs was investigated by qRT-PCR. The sequences of all primers are listed in Table S2. Whole-cell lysates from BMDCs were tested for the activation status of the NF-κB signaling pathway. The protein samples were probed by incubating with a 1:1000 dilution of anti-TLR4, anti-MyD88, anti-pp65, anti-p65, anti-IL-1β, anti-IL-6 or anti-β-actin antibodies overnight at 4℃. Then, donkey anti-mouse or anti-rabbit horseradish peroxidase-immunoglobulin G (1:5 000) antibodies were used to detect the signal of the corresponding primary antibody. Finally, the blots were visualized by an Amersham Imager 600 system with the Amersham ECL Western Blotting Detection Reagent.

### Analysis of OVA-specific CD8^+^ T cell responses in the spleen

C57BL/6J mice received three intravenous administrations of PBS, MC3 sLNPs-OVA, or SM-102 sLNPs-OVA (0.5 mg mRNA/kg body weight) via tail vein injection on days 0, 5, and 10. At the experimental endpoint (day 15), spleens and peripheral blood were harvested and processed into single-cell suspensions. Lysis of erythrocytes was performed with red blood cell lysis buffer, and splenocytes and PBMCs were stained with fluorophore-conjugated antibodies against CD45, CD4, CD8a, and OVA-tetramer for 30 min at 4 ℃. Viable cells were identified by DAPI exclusion. Antigen-specific CD8^+^ T cell populations (SIINFEKL-MHC I tetramer^+^/CD8^+^/CD45^+^) were quantified via flow cytometry. Data analysis was conducted in FlowJo software (V10).

### Tumor studies

In the B16F10-OVA therapeutic vaccination model, 6-8-week-old C57BL/6J mice were subcutaneously implanted with 5×10⁵ B16F10-OVA cells in the right flank. On days 8 and 13 post-tumor inoculation, the mice were immunized with PBS, MC3 sLNPs-OVA, or SM-102 sLNPs-OVA (with the dosages of mRNA at 0.5 mg/kg). Tumor growth was monitored using the established method. Mice were euthanized when tumor volume reached 1500 mm³. Tumor volume was measured every two days post-inoculation using a caliper and calculated with the formula *volume = ½ (length × width^2^)*, while tumor weight was recorded at the experimental endpoint. The tumors were dissected for further H&E staining and TUNEL cell apoptosis staining. The infiltration of CD4^+^ and CD8^+^ T cells in tumors was analyzed using flow cytometry and immunohistochemistry. In the B16F10-OVA pulmonary metastasis therapeutic model, 6-8-week-old C57BL/6 mice received 3×10⁵ B16F10-OVA cells via intravenous injection. PBS, empty SM-102 sLNPs, MC3 sLNPs-OVA, or SM-102 sLNPs-OVA (0.5 mg mRNA/kg) were administered intravenously on days 3, 8, and 13. Mice were euthanized on day 18 post-tumor inoculation, with lung metastasis assessed by lung weight measurement, total metastatic area quantification, and metastatic nodule counting.

### Apoptosis and cytotoxicity assessment in DC2.4 cells

To evaluate the cytotoxicity of mRNA-sLNPs, DC2.4 cells were plated in 96-well plates at a density of 1 × 10^4^ cells/well and allowed to adhere overnight. Following 24-hour exposure to sLNPs-OVA formulations, cellular viability was measured using a Cell Counting Kit-8 (CCK-8). Apoptosis was assessed in parallel with cytotoxicity measurements. Post-treatment DC2.4 cells were washed with PBS twice and resuspended in the Annexin V Binding Buffer (BioLegend, San Diego, CA, USA) at a density of 1×10^6^ cells/mL. Subsequently, aliquots of 100 μL cell suspension were transferred to 5 mL FACS tubes, followed by the addition of 2 µL APC Annexin V and 1 µL propidium iodide (PI). The mixture was gently vortexed and incubated for 15 minutes in the dark. Prior to flow cytometry acquisition, Binding Buffer was added to each tube, and samples were analyzed by flow cytometry.

### Investigation of preliminary safety

Following OVA-specific immune response assessments, C57BL/6J mice were euthanized and major organs such as heart, liver, spleen, lung, and kidney were surgically harvested. Tissues were immediately fixed with 4% paraformaldehyde (PFA) in PBS for 72 hours, then paraffin-embedded and transversely sectioned at 5 μm thickness. Histopathological evaluation of inflammatory responses was performed through H&E staining.

### Statistical analysis

Statistical significance was annotated directly in figure panels, with all analyses performed using GraphPad Prism v9.0. Data points represent values from each independent biological sample, with error bars corresponding to the standard error of the mean (SEM). Non-significant comparisons are labelled as "ns".

## Results

### MC3- and SM-102 sLNPs exhibit comparable physicochemical properties and encapsulation efficiency

**Figure 1:**
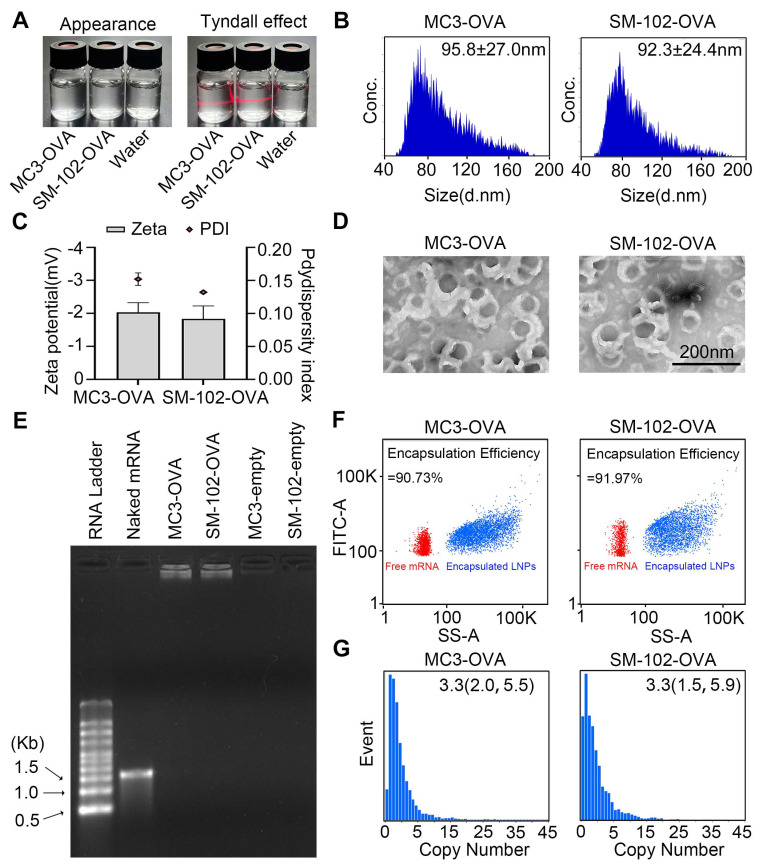
** Characterization of MC3 sLNPs and SM-102 sLNPs. (A)** Appearance and Tyndall effect of MC3 sLNPs-OVA, SM-102 sLNPs-OVA, and water. **(B)** Size distribution of MC3 sLNPs-OVA and SM-102 sLNPs-OVA as determined by NanoFCM. **(C)** Zeta potential and PDI of MC3 sLNPs-OVA and SM-102 sLNPs-OVA (n = 3 independent biological samples). **(D)** Representative TEM images of MC3 sLNPs-OVA and SM-102 sLNPs-OVA. Scale bar = 200 nm. **(E)** Agarose gel electrophoresis of naked mRNA, MC3 sLNPs-OVA, SM-102 sLNPs-OVA, empty MC3 sLNPs, and empty SM-102 sLNPs. **(F)** Encapsulation efficiency of OVA-mRNA by MC3 sLNPs and SM-102 sLNPs determined by NanoFCM. **(G)** Copy number of encapsulated mRNA in MC3 sLNPs-OVA and SM-102 sLNPs-OVA as determined by NanoFCM.

To fabricate a potent LNPs-based mRNA cancer vaccine without excessive proinflammatory properties, two FDA-approved ionizable phospho-lipids of SM-102 and DLin-MC3-DMA (MC3) were used to construct a spleen-targeted mRNA-LNPs vaccine with excellent clinical translation promise based on our previous report, and then tested for physicochemical properties and immunologic activity *in vitro* and *in vivo*. In brief, ionizable lipids of MC3 or SM-102, DSPC, cholesterol, DMG-PEG, and stearic acid dissolved in ethanol were rapidly mixed with mRNA dissolved in citrate buffer with the volume ratio of lipid to mRNA at 1:3. After replacing ethanol and citrate buffers with PBS, the MC3- and SM-102 sLNPs were obtained. Both MC3 sLNPs-OVA and SM-102 sLNPs-OVA formulations exhibited distinct Tyndall effects under laser illumination, confirming their stable nanoparticle dispersion in aqueous solution (Figure 1A). According to NanoFCM analysis, the diameters of MC3 sLNPs-OVA and SM-102 sLNPs-OVA were found to be comparable, with values of 95.8 ± 27.0 nm and 92.3 ± 24.4 nm, respectively. (Figure 1B). The near-neutral zeta potentials (-2 mV in PBS) suggested moderate electrostatic repulsion that likely contributed to colloidal stability without excessive aggregation, with narrow polydispersity indices (polydispersity index (PDI) ~0.15) indicative of homogeneous nanoparticle populations (Figure 1C). Representative transmission electron microscopy (TEM) imaging further corroborated these findings, demonstrating uniform spherical morphologies for both formulations with diameters consistent with NanoFCM measurements (Figure 1D). These collective physicochemical characteristics position both mRNA-sLNPs as promising candidates for efficient biological delivery, as optimal nanoparticle size (50-200 nm) and surface charge are critical determinants of cellular uptake and biodistribution.

For the encapsulation of mRNA, almost all of the mRNA was loaded within the nanoparticles, with no detectable free mRNA bands for either MC3- or SM-102 sLNPs-OVA, in contrast to the distinct migration of naked mRNA controls, as indicated by agarose gel electrophoresis (Figure 1E). Encapsulation efficiency (EE) values obtained for the MC3- and SM-102 sLNPs-OVA formulations depended markedly on the analytical platform. By the RiboGreen assay, MC3 sLNPs-OVA exhibited an EE of 83.6 ± 2.0 %, whereas SM-102 sLNPs-OVA reached 84.2 ± 3.3 % (Figure S1). In contrast, single-particle analysis on the NanoFCM yielded significantly higher values: 90.73% for MC3 sLNPs-OVA and 91.97% for SM-102 sLNPs-OVA (Figure 1F). The systematically higher EE values reported by NanoFCM relative to RiboGreen mirror documented differences in assay principle and sample handling. RiboGreen yields artificially depressed EE due to incomplete lysis efficiency, while NanoFCM provides a truer representation of LNP payload by directly profiling individual particles without lysis 36,37. Notably, both formulations exhibited comparable mRNA loading capacities, with average copy numbers of 3.3 mRNA molecules per nanoparticle and similar narrow distributions (MC3 sLNPs-OVA: 2.0-5.5; SM-102 sLNPs-OVA: 1.5-5.9) (Figure 1G). These results collectively demonstrate that both lipid formulations achieve efficient mRNA encapsulation while maintaining consistent payload characteristics, a critical prerequisite for ensuring dose uniformity in subsequent biological applications.

### SM-102 sLNPs enhance Splenic mRNA translation and DC antigen presentation with attenuated inflammation relative to MC3 sLNPs

**Figure 2:**
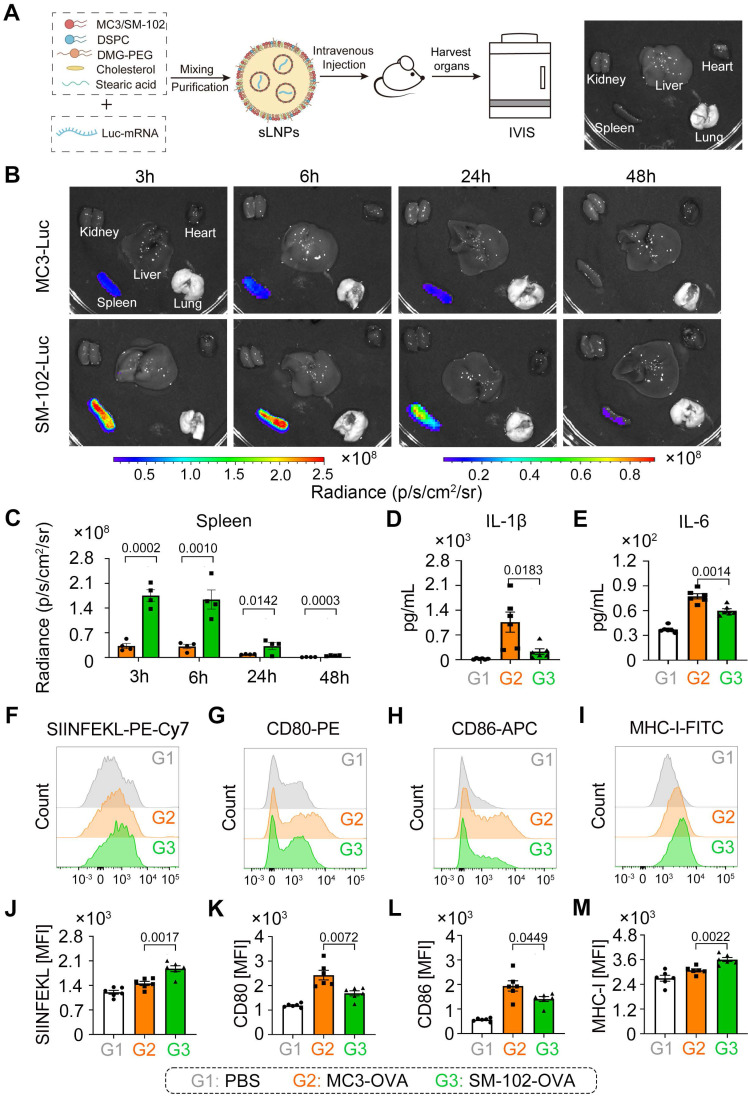
***In vivo* mRNA translation efficacy and pro-inflammatory properties of MC3- and SM-102 sLNPs. (A)** Schematic representation of the preparation of sLNPs and IVIS imaging. **(B)** *In vivo* translation of MC3- or SM-102 sLNPs-Luc in various organs (kidney, liver, lung, spleen, and heart) as visualized by IVIS imaging at 3, 6, 24, and 48 hours post i.v. injection. **(C)** Quantitative analysis of the radiance (p/s/cm²/sr) in the spleen for MC3- or SM-102 sLNPs-Luc at each time point (n = 4 independent biological samples).** (D, E)** Serum levels of pro-inflammatory cytokines IL-1β **(D)** and IL-6 **(E)** measured by ELISA 24 hours post treatment with PBS (G1), MC3-sLNPs-OVA (G2), or SM-102-sLNPs-OVA (G3) (n = 6 independent biological samples). **(F-M)** Flow cytometry analysis of DC activation markers in spleens 24 hours post treatment with different formulations. Representative histograms and quantification of mean fluorescence intensity (MFI) for MHC-I bound to SIINFEKL peptide (H-2K^b^-SIINFEKL) **(F, J)**, CD80**(G, K)**, CD86 **(H, L)** and MHC-I (H-2K^b^) (I, M) on CD11c^+^ DCs (n = 6 independent biological samples). Statistical significance was determined by two-way ANOVA with Tukey' s test for **(C)** and one-way ANOVA with Tukey' s multiple comparisons test for **(D)**, **(E)**, and (J-M).

The formulation process for both MC3- and SM-102 sLNPs involved optimized encapsulation of Luc-mRNA with auxiliary lipids, followed by purification and systemic administration in mice, with subsequent organ-specific imaging via the IVIS system (Figure 2A). Both formulations exhibited pronounced spleen-targeting tendencies, with splenic tissue displaying markedly stronger bioluminescent signals than other major organs (liver, lung, kidney, and heart), as observed through IVIS imaging analysis (Figure 2B). Notably, the spleen exhibited higher radiance signals with SM-102 sLNPs-Luc compared to MC3-sLNPs-Luc, whereas both formulations demonstrated similarly low fluorescence intensities in hepatic and pulmonary tissues. The luciferase protein exhibited rapid *in vivo* translation, evidenced by detectable bioluminescence in splenic tissue at 3 hours post-administration. Moreover, bioluminescence signals persisted for approximately 48 h, indicating the duration of antigen expression *in vivo* (Figure 2C). Specifically, SM-102 sLNPs-Luc demonstrated superior splenic targeting, with a higher percentage of Luc-mRNA translation localized to the spleen compared to the MC3 formulation (Figure S2A), suggesting that structural differences in SM-102 may enhance nucleic acid release kinetics or translational compatibility within splenic microenvironments. According to the experimental results, the transfection efficacy of mRNA exhibits sustained stability when stored at 4°C for a minimum duration of 14 days (Figure S2B-C). This observation gains significance when compared to conventional LNPs. While MC3- and SM-102-based LNPs (composed of MC3/SM-102, cholesterol, DSPC and DMG-PEG) exhibit enhanced liver selectivity, the SM-102 formulation demonstrated enhanced Luc mRNA translation in the spleen and liver than its MC3-based counterpart (Figure S3A-B). This dual enhancement indicates that the improved mRNA translation by SM-102 compared to MC3 operates through mechanisms independent of stearic acid-doping enabled spleen selectivity.

DC maturation and cytokines of IL-1β and IL-6 in the serum of mice treated with different formulations for 24 h were investigated. According to the experimental results, serum cytokines of IL-1β and IL-6 in mice treated with SM-102 sLNPs-OVA were less than those of the MC3 sLNPs group (Figure 2D-E). Moreover, the enhanced antigen presentation, as indicated by elevated levels of MHC-I and MHC-I bound to SIINFEKL complex and limited co-stimulatory molecules of CD80 and CD86 on splenic DCs from the SM-102 sLNPs group than the MC3 sLNPs group, was observed (Figure 2F-M).

### SM-102 sLNPs demonstrate enhanced lysosomal escape over MC3 nanoparticles despite identical cellular uptake

**Figure 3:**
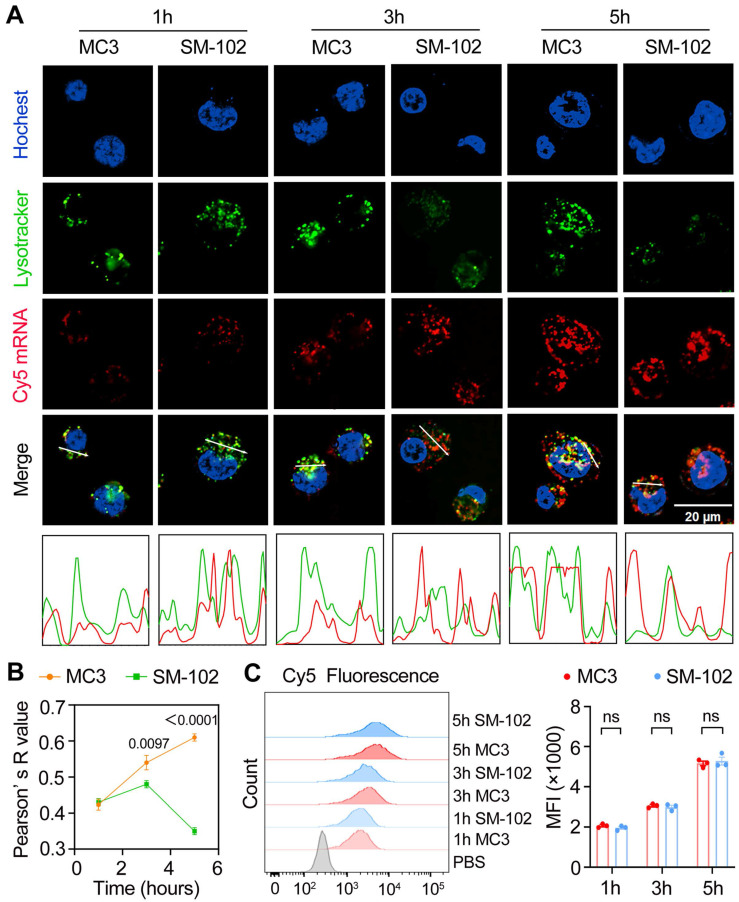
** Temporal dynamics of cellular uptake and intracellular trafficking of MC3- and SM-102 sLNPs. (A)** Confocal microscopy images of DC2.4 cells treated with MC3 and SM-102 sLNPs encapsulating Cy5 mRNA at 1, 3, and 5 hours. Lysosomes were stained with LysoTracker Green, the nuclei were labeled with Hoechst, and Cy5 mRNA (red) was visualized in red. Scale bar: 20 μm. The merged images show the co-localization of the nanoparticles with lysosomes. The intensity profiles below the images represent the fluorescence intensity of LysoTracker (green) and Cy5 mRNA (red) along the white lines in the merged images. **(B)** Pearson's correlation coefficient (R value) between the fluorescence of LysoTracker and Cy5 mRNA over time, indicating the co-localization of the nanoparticles with lysosomes (n = 3 independent biological samples). **(C)** Flow cytometry analysis of Cy5 fluorescence (MFI) in DC2.4 cells treated with MC3 and SM-102 nanoparticles at 1, 3, and 5 hours (n = 3 independent biological samples). Statistical significance was determined by two-way ANOVA with Tukey' s test for **(B)** and **(C)**.

At the 1 h time point, both nanoparticle formulations exhibited similar lysosomal trafficking behaviors in DC2.4 cells, as visualized through colocalized Cy5-mRNA and LysoTracker signals in confocal micrographs (Figure 3A). It has been reported that different degrees of lysosomal destruction in cells could be observed after being treated with nanoparticles composed of different ionizable lipids 38,39. The differences in lysotracker signal intensity in the SM-102 group and MC3 group 3 h and 5 h post-treatments indicated that the lysosome disruption ability of LNPs composed of SM102 was stronger than that of LNPs composed of MC3, which was consistent with previous literature. Besides, SM-102 sLNPs-Cy5 exhibited markedly reduced co-localization with lysosomes compared to MC3 sLNPs-Cy5 at 3 h and 5 h. This trend was corroborated quantitatively by Pearson' s correlation analysis (Figure 3B), where SM-102 sLNPs-Cy5 displayed significantly lower R-values than MC3 sLNPs-Cy5 at these intervals, indicative of enhanced lysosomal escape efficiency for the SM-102 formulation. Notably, the intensity profiles in Figure 3a showed spatially distinct fluorescence peaks for SM-102 sLNPs-Cy5 relative to lysosomal signals at prolonged incubation times, further supporting diminished entrapment in acidic compartments.

In contrast to the differential lysosomal trafficking, no statistically significant differences in Cy5 fluorescence intensity between MC3- and SM-102 sLNPs-treated cells were observed across all timepoints according to the kinetics analyses of cellular uptake by flow cytometry and confocal microscopy (Figure 3C and Figure S4). This parity in total nanoparticle internalization suggests that the observed superiority in lysosomal escape by SM-102 sLNPs-Cy5 is not attributable to differences in cellular uptake capacity. Rather, the data collectively imply that SM-102 sLNPs-Cy5 possess intrinsic physicochemical properties favoring endosomal membrane destabilization or trafficking pathway modulation, thereby facilitating earlier and more efficient cytosolic delivery of mRNA cargo compared to MC3 counterparts.

For cytotoxicity assessment, the cell viability of DC2.4 cells treated with MC3 or SM-102 sLNPs-OVA at increasing mRNA concentrations (0-2000 ng/mL), measured by CCK-8 assay. Both compounds showed no significant changes in cell viability across the tested concentrations, and no statistically significant differences in cytotoxicity were observed between the two groups. Besides, similar apoptotic rates were observed for MC3- and SM-102 groups, with no significant differences between the two compounds (Figure S5A-B).

### SM-102-sLNPs enhance antigen presentation but attenuate inflammatory responses via TLR4-NF-κB lower activation compared to MC3-sLNPs

**Figure 4:**
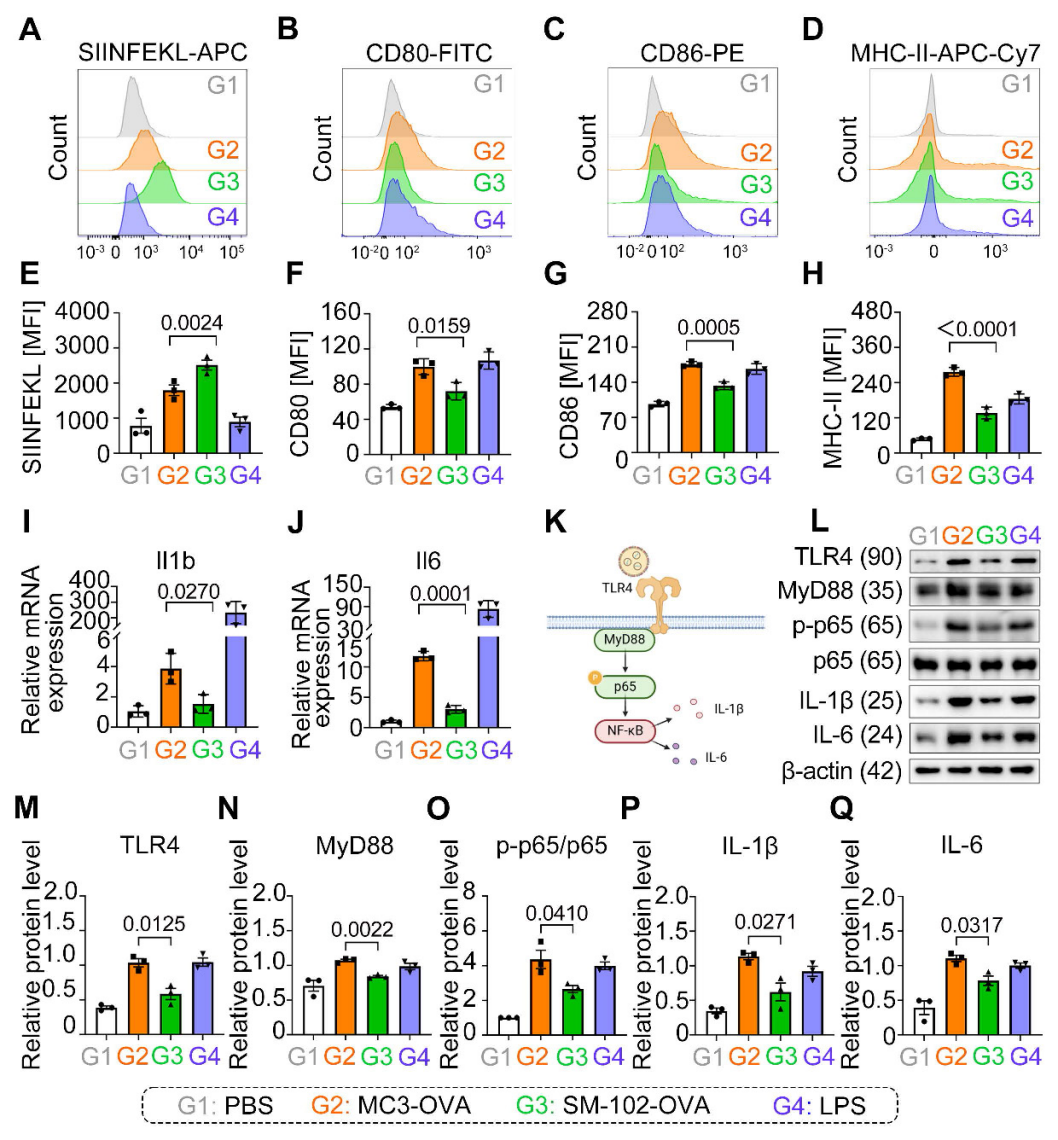
** Analysis of Antigen Presentation and Inflammatory Responses in BMDCs Treated with MC3- and SM-102 sLNPs-OVA. (A-D)** Flow cytometry analysis of SIINFEKL-APC **(A)**, CD80-FITC **(B)**, CD86-PE **(C)**, and MHC-II-APC-Cy7 **(D)** expression in BMDCs treated with PBS (G1), MC3 sLNPs-OVA (G2), SM-102 sLNPs-OVA (G3), or LPS (G4). **(E-H)** Quantitative analysis of MFI of H-2K^b^ bound to SIINFEKL **(E)**, CD80 **(F)**, CD86 **(G)**, and MHC-II **(H)** in the respective groups (n = 3 independent biological samples). **(I-J)** Relative mRNA expression of IL-1β **(I)** and IL-6 **(J)** in the treated BMDCs (n = 3 independent biological samples). **(K)** Schematic diagram of the innate immune activation of LNPs-based mRNA vaccine through TLR4-NF-κB signaling pathway (Created in https://BioRender.com ). **(L)** Western blot analysis of TLR4, MyD88, p65, p-p65, IL-1β, IL-6, and β-actin in BMDCs treated with different formulations. **(M-Q)** Relative protein levels of TLR4 (M), MyD88 (N), p-p65/p65 **(O)**, IL-1β **(P)**, and IL-6 **(Q)** in the respective groups (n = 3 independent biological samples). Statistical significance was determined by one-way ANOVA with Tukey' s multiple comparisons test for **(E-J)** and **(M-Q)**.

SM-102 sLNPs-OVA significantly enhanced SIINFEKL presentation compared to MC3 sLNPs-OVA (Figure 4A, E), yet paradoxically reduced surface expression of co-stimulatory markers CD80, CD86, and MHC-II (Figure 4B-D, F-H).

Our results show that SM-102/MC3 sLNPs-OVA-treated BMDCs not only exhibited significant upregulation of IL-1β and IL-6 mRNA transcription (Figure 4I, J), but also elevated protein expression levels of these pro-inflammatory cytokines (Figure 4L, P, Q), accompanied by activation of the TLR4-MyD88-NF-κB signaling pathway. Notably, compared to its marked enhancement of antigen presentation capacity, SM-102 sLNPs -OVA vaccine induces relatively weaker pro-inflammatory responses (Figure 4I-Q). This decoupling phenomenon between enhanced antigen presentation and decreased co-stimulatory expression with attenuated pro-inflammatory effectors suggests that sLNPs with reduced inflammation may exert potent mRNA vaccine delivery potential but negligible security risks.

The immunomodulatory properties of SM-102 mRNA-sLNPs vaccine were fundamentally linked to their lipid composition rather than antigen engagement, evidenced by empty SM-102 formulations (without OVA) demonstrating equivalent downregulation of CD80/CD86/MHC-II surface markers (Figure S6A-C) and transcriptional suppression of IL-1β/IL-6 (Figure S6D- E) when benchmarked against MC3 controls. The consistent downregulation of co-stimulatory markers and cytokines across both antigen-loaded and empty SM-102 formulations (Figure 4B-D, Figure S6A-E) strongly implicates SM-102's lipid architecture in blunting MyD88-dependent TLR4-NF-κB signaling, a pathway central to DCs' maturation and inflammatory responses. Notably, while SM-102 enhanced antigen processing/stability as evidenced by elevated SIINFEKL presentation (Figure 4E), its limited effects on DC activation markers and cytokine production suggest a decoupling of antigen presentation efficiency from inflammatory pathway engagement. These findings underscore a formulation-dependent divergence, with SM-102 sLNPs uniquely balancing enhanced antigen presentation against attenuated immunostimulation-a functional contrast to MC3 sLNPs that positions them as strategically favorable for controlled immunity applications.

### SM-102 sLNPs-OVA elicit enhanced systemic and antigen-specific T cell responses compared to MC3 sLNPs-OVA

**Figure 5:**
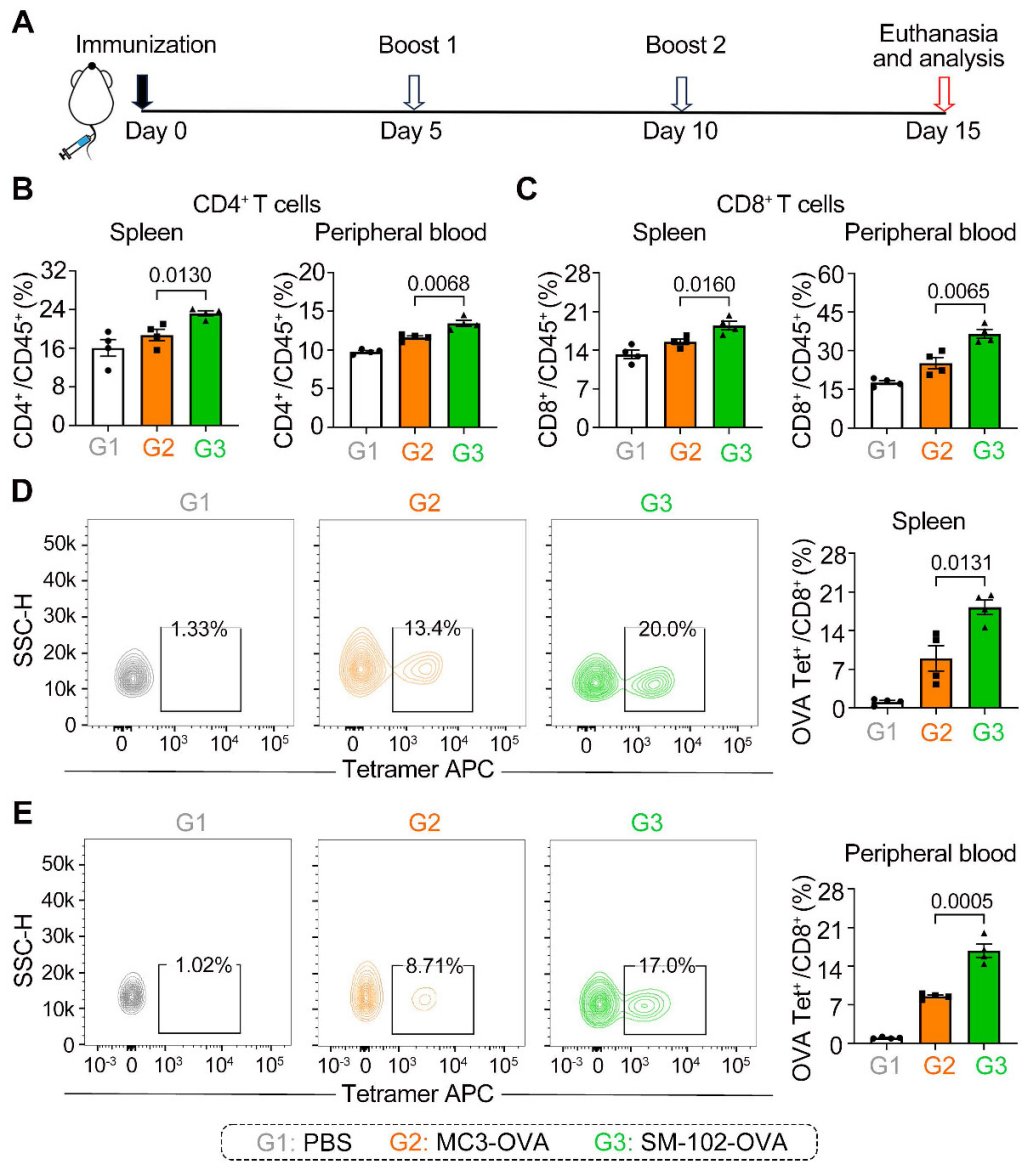
** Differential induction of T cell subsets and antigen-specific responses by MC3 and SM-102 sLNPs-OVA immunization in mice. (A)** Schematic representation of the immunization schedule. **(B, C)** Frequencies of CD4^+^ and CD8^+^ T cells in the spleen **(B)** and peripheral blood **(C)** of mice immunized with PBS, MC3 sLNPs-OVA, or SM-102 sLNPs-OVA. Data are expressed as the percentage of CD4^+^ or CD8^+^ cells among CD45^+^cells (n = 4 independent biological samples). **(D, E)** Flow cytometric analysis of OVA-specific CD8^+^ T cells in the spleen **(D)** and peripheral blood **(E)** of immunized mice. The percentage of OVA tetramer^+^ CD8^+^ T cells is shown (n = 4 independent biological samples). Statistical significance was determined by one-way ANOVA with Tukey' s multiple comparisons test for **(B-E)**.

To assess the antigen-specific T cell responses elicited by MC3 and SM-102 sLNPs-OVA, mice were immunized and boosted following the schedule in Figure 5A, with immune profiling conducted on day 15. It should be noted that each injection was administered at an mRNA vaccine dose of 0.5 mg/kg. The two LNP formulations exhibited distinct patterns of T cell subset induction, as quantified in the B16F10-OVA subcutaneous tumor model. Mice immunized with SM-102 sLNPs-OVA exhibited elevated frequencies of CD4^+^ and CD8^+^ T cells in both peripheral blood and splenic tissues compared to those receiving MC3 sLNPs-OVA (Figure 5B-C). These results demonstrate that SM-102 sLNPs-based mRNA vaccine evokes systemic cellular responses more effectively than MC3-based formulations.

Antigen-specific CD8^+^ T cell responses were further evaluated using OVA tetramer staining. SM-102 sLNPs-OVA immunization generated a higher proportion of OVA-specific CD8^+^ T cells in both the spleen and peripheral blood relative to MC3 sLNPs-OVA (Figure 5D-E). The increased antigen-specific response aligns with the broader CD8^+^ T cell expansion observed in SM-102 sLNPs-OVA-immunized mice, implying that SM-102 sLNPs may promote stronger MHC-I antigen presentation or more efficient cross-priming of cytotoxic T lymphocytes. Moreover, histopathological examination (H&E staining) of various organs at the experimental endpoint showed no significant organic damage at this dosage level (Figure S8). These differential outcomes highlight the critical role of lipid nanoparticle composition in shaping cellular immunity, with SM-102 sLNPs-OVA demonstrating superior capacity to drive both polyclonal and antigen-focused T cell activation.

### SM-102 sLNPs-OVA outperforms MC3 sLNPs-OVA in tumor suppression and metastasis inhibition via enhanced T-cell infiltration and apoptosis induction

**Figure 6:**
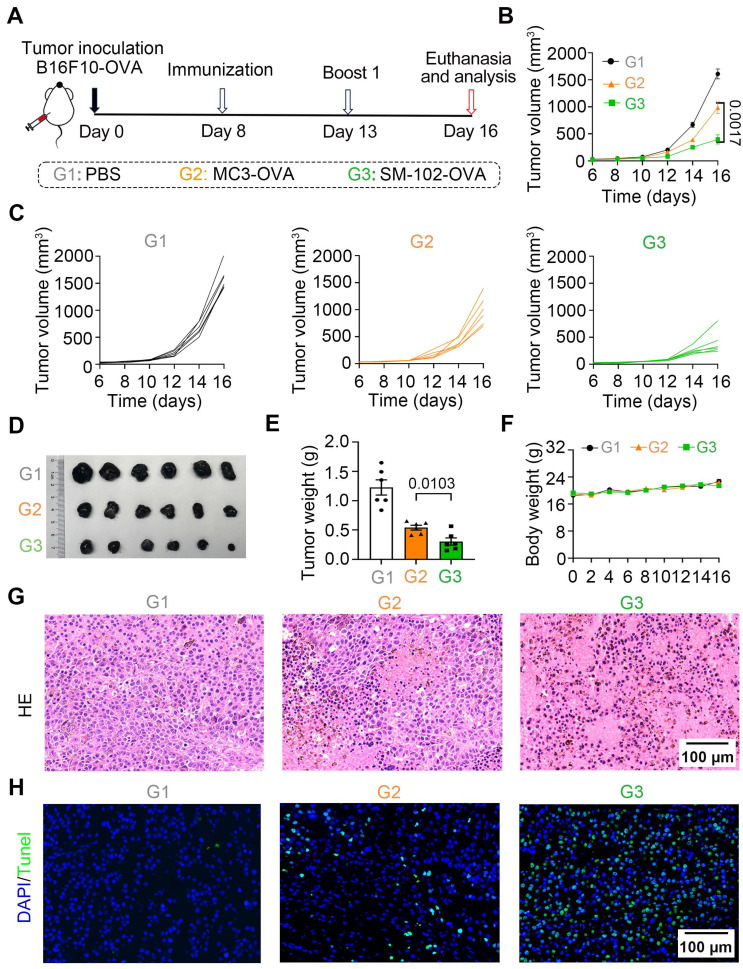
** Evaluation of tumor growth and histopathological changes in B16F10-OVA tumor-bearing mice treated with PBS, MC3 sLNPs-OVA, or SM-102 sLNPs-OVA. (A)** Schematic timeline of the experimental procedure: tumor inoculation (Day 0), immunization (Day 8), first boost immunization (Day 13), and termination (Day 16). **(B)** Tumor volume dynamics over time (n = 6 independent biological samples). **(C)** Individual tumor volume trajectories for each group. **(D)** Tumor photographs from PBS (control), MC3-, and SM-102 treated groups. **(E)** Tumor weight comparison across groups (n = 6 independent biological samples). **(F)** Body weight comparison across groups (n = 6 independent biological samples). **(G)** H&E-stained tumor sections. **(H)** TUNEL (green fluorescence) staining for apoptosis. Scale bar = 50 μm. Statistical significance was determined by two-way ANOVA with Tukey' s test for **(B)** and one-way ANOVA with Tukey' s multiple comparisons test for **(E)**.

To investigate the efficacy of different interventions in inhibiting the growth of tumor *in vivo*, the therapeutic caner immunotherapy experiment was performed following the timeline protocol (Figure 6A). As shown by the tumor growth curves, SM-102-treated groups of B16F10-OVA tumor-bearing mice exhibited significant suppression of tumor growth compared to the PBS and MC3 groups (Figure 6B-C). At the endpoint of the experiment, tumor volume (Figure 6D) and weight (Figure 6E) were markedly reduced in the SM-102-based vaccine than its MC3-based counterpart. Moreover, body weight did not exhibit significant alterations in any of the treatment groups before the study endpoint, with no statistically discernible differences observed between groups, collectively indicating favorable treatment safety profiles (Figure 6F). Besides, the tumor tissues were sliced and stained with hematoxylin-eosin and TUNEL. Among the three groups, decreased tumor cellularity and increased apoptosis were observed in SM-102 sLNPs-OVA-treated tumors than the other two groups (Figure 6G-H). These data collectively indicate that SM-102 sLNPs-OVA exerts enhanced tumor immunotherapy efficacy than MC3 sLNPs-OVA.

**Figure 7:**
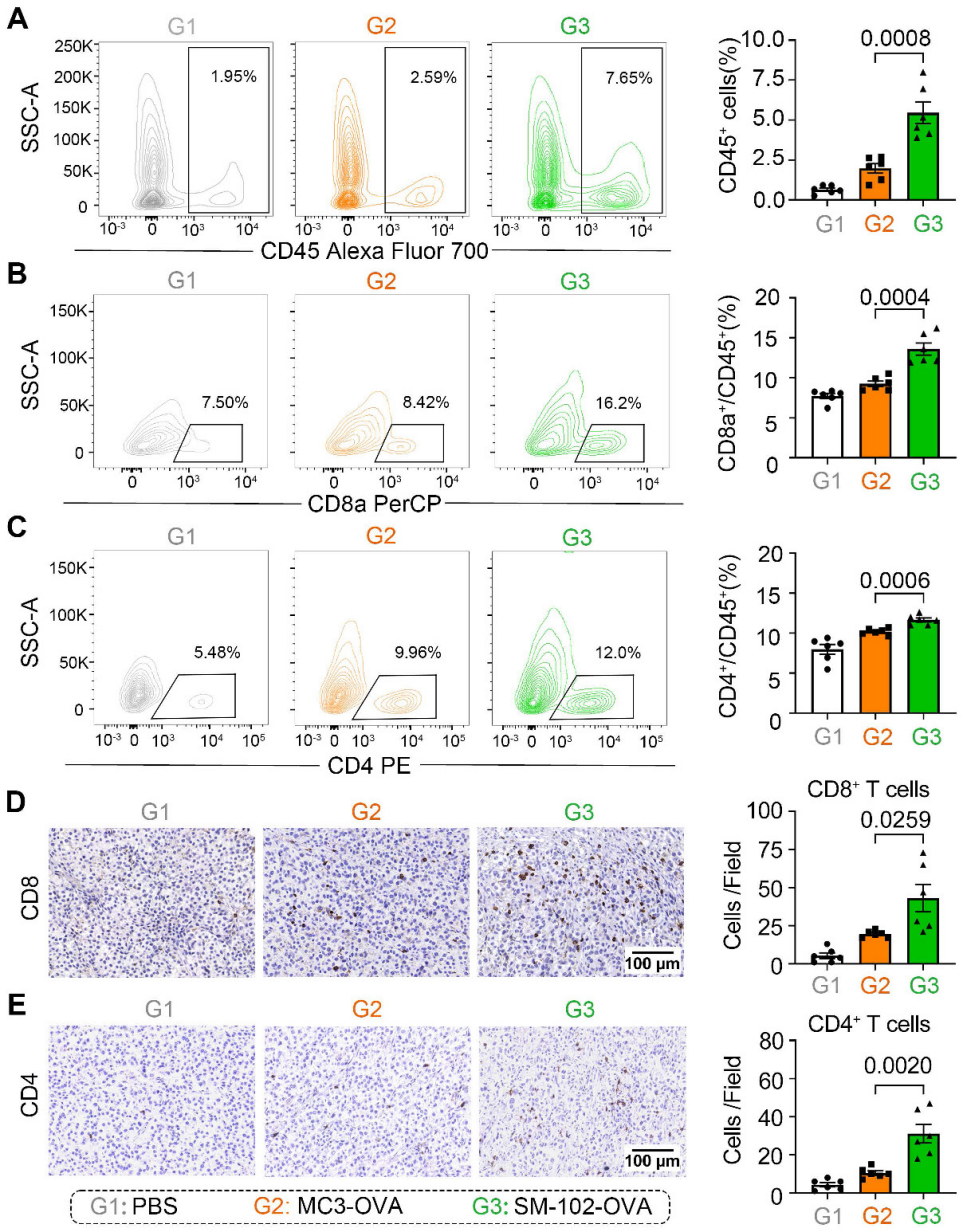
** Immune cell infiltration analysis in tumor tissues from PBS, MC3 sLNPs-OVA, and SM-102 sLNPs-OVA treated mice. (A-C)** Quantification of CD45^+^ cells **(A)**, CD8a^+^ cells **(B)**, and CD4^+^ cells **(C)** in tumor tissue by flow cytometry at the end of the study (n = 6 independent biological samples). Representative immunohistochemical staining pictures and quantification of CD8^+^ T cells **(D)** and CD4^+^ T cells **(E)** in tumor tissue (n = 6 independent biological samples). Scale bar = 100 μm. Statistical significance was determined by one-way ANOVA with Tukey' s multiple comparisons test for **(A-E)**.

Enhanced apoptosis of tumor cells in SM-102 sLNPs-OVA treated tumor tissues stimulates us to investigate the changes of tumor-killing immune cells accumulation in the tumor microenvironment. According to the results of flow cytometry, elevated infiltration of CD45^+^ leukocytes, CD8a^+^ T cells, and CD4^+^ T cells in SM-102 sLNPs-OVA treated tumors than MC3 sLNPs-OVA treated tumors was observed (Figure 7A-C, S9). The increased CD8^+^ and CD4^+^ T cell densities in the SM-102 group than the MC3 group were also confirmed by immunohistochemistry (Figure 7D-E). Furthermore, the activation of T cells was assayed by flow cytometry through the expression levels of CD69 on T cells. SM-102-treated groups exhibited higher CD69 levels in CD8^+^ and CD4^+^ T cells than PBS and MC3 controls (Figure S10A-B), indicating a preferable status of T cells for tumor killing. Concurrently, SM-102 sLNPs-OVA demonstrated a robust capacity to remodel the tumor microenvironment, with particular enhancement of T cell infiltration and activation.

**Figure 8:**
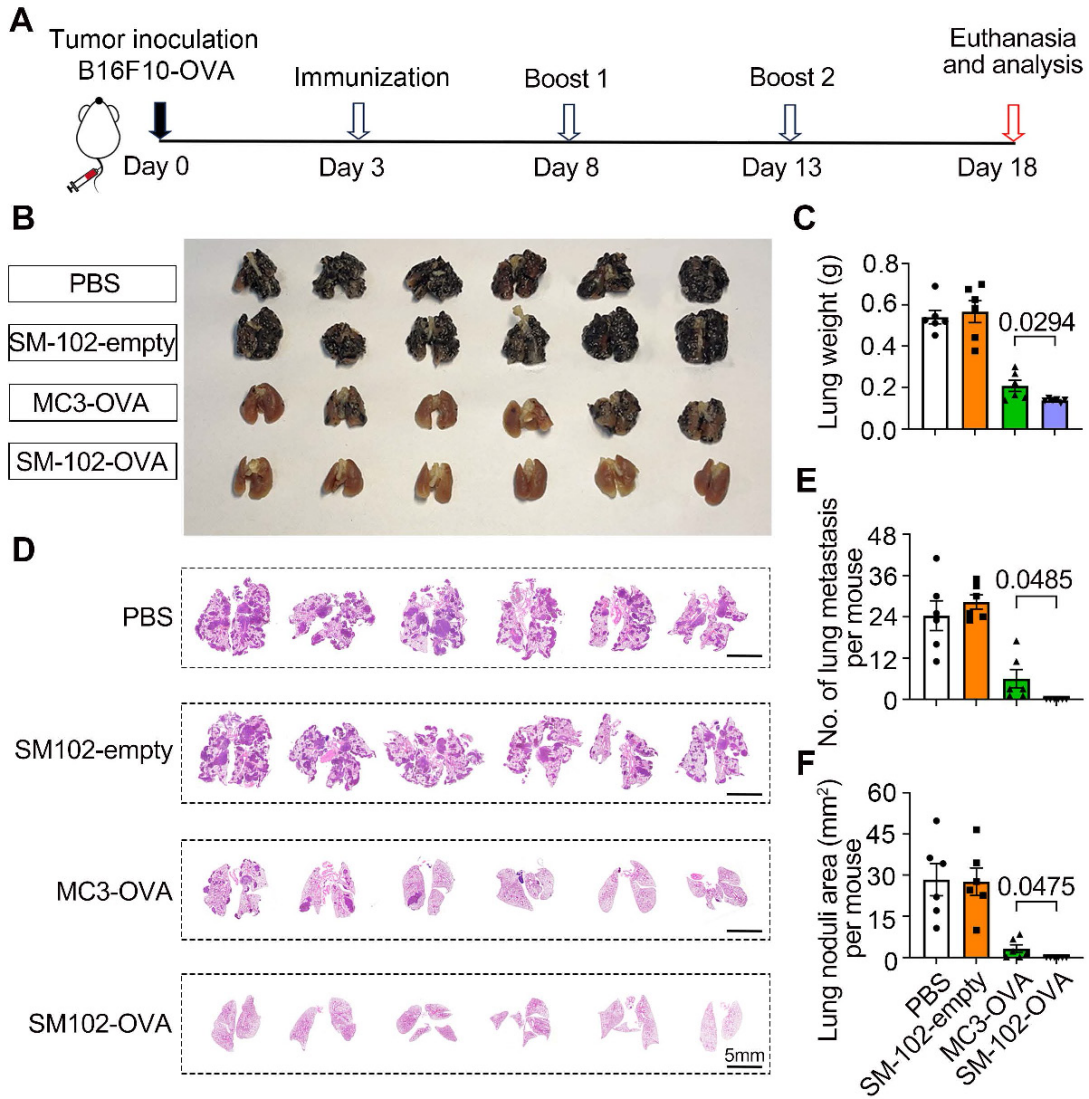
** Evaluation of metastatic suppression in PBS, empty SM-102-sLNPs, MC3 sLNPs-OVA, and SM-102 sLNPs-OVA treatment groups. (A)** Schematic timeline of the experimental design. B16F10-OVA tumor cells were inoculated on Day 0, followed by immunization (Day 3), two booster immunizations (Days 8 and 13), and termination of the experiment (Day 18). **(B)** Macroscopic images of lungs from each treatment group. **(C)** Analysis of lung weight at the end of the study (n = 6 independent biological samples). **(D)** Histological sections of lung tissues were stained to visualize tumor nodules. **(E)** Quantification of metastatic lung nodules (n = 6 independent biological samples). **(F)** Analysis of total tumor nodule area (n = 6 independent biological samples). Statistical significance was determined by one-way ANOVA with Tukey' s multiple comparisons test for **(C)**, **(E)**, and **(F)**.

To evaluate the efficacy of different treatments in suppressing the experimental lung metastasis following intravenous injection of B16F10-OVA cells, mice were subjected to the experimental timeline outlined in Figure 8A. Macroscopic examination of lungs (Figure 8B) revealed that PBS-treated mice exhibited dense black tumor nodules, indicative of advanced metastasis. In contrast, reduced nodule size and number in lungs from SM-102-OVA and MC3-OVA groups were observed, with the SM-102-OVA group showing near-complete resolution of visible lesions (Figure 8B). The findings were further corroborated through lung weight measurements. The PBS group exhibited the highest lung weight, while the SM-102-OVA group treatment significantly reduced lung weight, reflecting diminished tumor burden (Figure 8C). Extensive tumor metastasis was observed in PBS-treated lungs, whereas SM-102-OVA-treated tissue displayed minimal pathological changes, as confirmed by histological evaluation (Figure 8D). Statistically, fewer metastatic nodule counts (Figure 8E) and smaller total tumor nodule area (Figure 8F) in the SM-102-OVA group than the MC3-OVA group were further confirmed. Immunohistochemical staining of lung tissues revealed distinct patterns of T cell infiltration across treatment groups. As a result, both spleen-selective LNPs-based mRNA vaccine formulations induced greater CD8^+^ and CD4^+^ T cell accumulation compared to PBS controls (Figure S11A-B), indicating that the superior cancer immunotherapy efficacy may be attributed to the changes of microenvironment with enhanced immune cells infiltration. These results collectively demonstrate that SM-102-OVA treatment significantly inhibits tumor progression and metastasis, outperforming MC3-OVA formulations.

## Discussion

LNPs-based nanotechnology has made great progress in RNA delivery 40, as siRNA-loaded LNPs (Onpattro®) and mRNA-loaded LNPs (mRNA-1273) have been approved for different diseases in the clinic by the FDA, in recent years. To fabricate a potent LNPs-based mRNA cancer vaccine without excessive proinflammatory properties, two FDA-approved ionizable phospholipids of SM-102 and MC3 were used to construct a spleen-targeted mRNA-LNPs vaccine with excellent clinical translation promise based on our previous report 23, and then tested for physicochemical properties and immunologic activity *in vitro* and *in vivo*. It has been demonstrated that SM-102 sLNPs with moderated inflammatory properties enhanced mRNA translation efficiency and thereby induced stronger antigen-specific T cell responses compared to the MC3-formulated counterparts, while simultaneously reducing proinflammatory cytokine induction. The counterintuitive findings of reduced immunogenicity correlate with enhanced antigen-presenting efficacy and cellular responses might challenge the conventional paradigm that equates stronger adjuvant effects with better vaccine potency.

Recent studies have demonstrated that subtle variations in the chemical architecture of ionizable lipids, including alkyl chain branching and headgroup polarity, can substantially alter mRNA translation efficiency in specific organs mediated by LNPs 41-44. For instance, the ionizable lipid 5A2-Sc8 enhances pulmonary mRNA translation through charge modulation 41, while DLin-KC2-DMA improves hepatocyte protein translation via pKa optimization 42. In this study, we report that within a stearic acid-modified LNPs previously optimized for spleen-selective mRNA delivery, substitution of MC3 with SM-102 significantly amplified splenic mRNA translation efficiency. This finding suggests that structural refinement of ionizable lipids can achieve secondary amplification of mRNA translation in target organs through modulation of nucleic acid release kinetics or translational microenvironment compatibility, even after tissue tropism has been established. Emerging evidence indicates that such enhancement may correlate with precise regulation of lysosomal escape mechanisms 45. Systematic comparisons by Han et al. revealed that multi-tailed ionizable lipids with increased tail cross-sectional area form pronounced conical structures, with this geometric deformation significantly enhancing fusion efficiency between lipid bilayers and endosomal membranes 46. Furthermore, Mitchell and colleagues employed directed chemical evolution to demonstrate that asymmetric branching in ionizable lipids induces curvature changes upon protonation, optimizing interactions with endosomal phospholipids to facilitate lysosomal escape 47. Notably, our study observed comparable cellular uptake between SM-102 sLNPs and MC3 sLNPs in dendritic cells, yet SM-102 formulations exhibited superior lysosomal escape efficiency. These results collectively indicate that within organ-targeted LNP systems, structural optimization of ionizable lipids can enhance mRNA translation through specific improvement of lysosomal escape efficacy, independent of alterations in upstream delivery pathways.

The mRNA-LNP vaccine initiates adaptive immune responses by synergistically activating the primary signal (antigen-specific signal) and secondary signal (co-stimulatory signal) for potent cancer immunotherapy 48,49. The core of the primary signal lies in DCs processing antigens and presenting them via MHC molecules to T cells, thereby triggering antigen-specific immune responses. Endogenously synthesized antigens by mRNA tends to form peptide-MHC class I complexes (p-MHC I), which evokes antigen-specific CD8⁺ T cell responses 50,51. Thus, MHC-I levels on splenic dendritic cells were investigated. According to the experimental results, the expression of MHC-I on splenic dendritic cells from mice treated with SM-102 sLNPs was significantly enhanced than that of the MC3 sLNPs group. It has been reported that the level of p-MHC I shapes the magnitude of specific CD8⁺ T cell responses, which is essential for cancer immuno-therapy 52. The enhanced expression of MHC-I on splenic dendritic cells in SM-102 sLNPs indicated its stronger ability to induce CD8⁺ T cell response. Notably, the high-efficiency expression of antigen mRNA directly increases intracellular antigen protein synthesis. Higher antigen protein levels provide more substrates for antigen processing, potentially enhancing the efficiency of antigen presentation 53,54. This aligns with our findings that SM-102-incorporated sLNPs exhibit concurrent increases in both expression efficiency and antigen presentation efficiency compared to MC3-based sLNPs, indicating that SM-102 sLNPs significantly enhances DCs' antigen processing and presentation capabilities by optimizing OVA mRNA translation levels. *In vivo* experiments further revealed that this vaccine preferentially induced a greater number of OVA-specific T cells in the spleen. Consequently, this amplifies the initiation efficiency of the primary signal (MHC-antigen-TCR pathway).

The secondary signal of adaptive immune responses is mediated by interactions between co-stimulatory molecules (CD80/CD86, CD40, etc.) on DCs and receptors (CD28, CD40L, etc.) on T cells, serving as an essential requirement for full T-cell activation 55. During this process, activation of the TLR4-NF-κB signaling pathway concurrently promotes DC maturation, co-stimulatory molecule expression, and pro-inflammatory cytokine secretion 56. Traditional adjuvants like LPS amplify co-stimulatory signals through TLR4 hyperactivation mechanisms but often induce significant toxic effects 57. Recent studies reveal that ionizable lipid components in LNPs exhibit adjuvant-like effects by directly activating innate immune signaling pathways independently of nucleic acid payload, though they may trigger inflammation-related side effects 17,20,58,59. Molecular docking analyses demonstrate structural similarities between the tertiary amine groups in ionizable lipid molecules and the glucosamine backbone of LPS, potentially facilitating their specific binding to the TLR4/MD-2 complex 17. Our experimental data demonstrate that SM-102/MC3 sLNPs-treated BMDCs exhibit not only significant upregulation of IL-1β and IL-6 mRNA transcription, but also elevated protein expression levels of these pro-inflammatory cytokines, accompanied by activation of the TLR4-MyD88-NF-κB signaling pathway. Notably, compared to its marked enhancement of antigen presentation capacity, SM-102 sLNPs-based mRNA vaccine induces relatively weaker pro-inflammatory responses. This decoupling phenomenon between enhanced antigen presentation and decreased co-stimulatory expression with attenuated pro-inflammatory effectors suggests that sLNPs with reduced inflammation may exert potent mRNA vaccine delivery potential but negligible security risks.

The clinical efficacy of tumor immunotherapy critically depends on the infiltration level of cytotoxic immune cells within the tumor microenvironment 60,61. In the B16F10-OVA melanoma model, the SM-102-formulated sLNPs vaccine demonstrated superior efficacy compared to its MC3-based counterparts, eliciting significantly stronger antigen-specific cellular immune responses. Furthermore, histological analysis revealed substantial tumor-infiltrating T lymphocyte accumulation in SM-102 sLNPs-treated subjects, indicating enhanced immune cell recruitment to tumor sites. Moreover, the expression of the activation marker of CD69 on CD8^+^ T cells in the tumor tissue of mice from SM-102 sLNPs group was also significantly increased compared to the MCs sLNPs group despite a decrease in CD80/CD86 expression on splenic DCs, indicating that lower inflammatory vectors have stronger potential for mRNA translation and cellular immune response than much higher inflammatory vectors, which was consistent with previous reports 32,62-64. The synergistic effects formed among the three hallmark features of this vaccine system—enhanced antigen presentation capacity, limited co-stimulatory molecule expression, and controlled inflammatory responses—represent a major breakthrough for mRNA-LNP technology in the field of cancer immunotherapy.

The use of the OVA as a model antigen, while standard in vaccine research, does not fully reflect the complexity of neoantigen encountered in the real-world of tumor development. Delivering neoantigen or tumor-associated antigen that better mimics clinical conditions in other animal models, such as non-human primates, would advance this proof-of-concept toward clinical translation in cancer immunotherapy. Other ionizable lipids such as C12-200 and ALC-0315 were also extensively investigated in mRNA-based gene therapy or vaccine development 65-67. As the mRNA delivery efficacy and pro-inflammatory properties of LNPs come from their ionizable lipid component 17,18, other ionizable lipids might also be suited for this proof-of-concept, which remains to be investigated following the current procedure in future studies. In this study, natural long-chain saturated fatty acid-modified mRNA-LNPs were intravenously injected into mice, which could selectively deliver mRNA to the spleen and initiate spleen-mediated systemic immune responses for tumor immunotherapy. According to previous reports, the administration routes could affect the *in vivo* distribution and translation of mRNA-based nanomedicines. In brief, nanoparticles preferentially accumulate in lymph nodes when subcutaneously injected 68,69, while they preferentially accumulate in organs such as the pancreas in the abdominal cavity when intraperitoneally injected 70. In future work, different administration routes of our formulations might be applied *in vivo* for different experimental purposes to expand their biomedical application to more diseases.

In this study, we engineer a spleen-selective LNPs-based mRNA vaccine by decoupling the inflammation from cellular immunity mediated cancer immunotherapy and elucidate its potential mechanisms as the molecular structure of ionizable lipid significantly enhances antigen expression and presentation efficiency by improving lysosomal escape efficiency while simultaneously attenuating TLR4-mediated pro-inflammatory signaling activation. This decoupling of potent cellular responses from highly inflammatory LNPs-based vaccine-associated innate immune activation breaks through the traditional adjuvant paradigm reliant on strong immune activation. The spleen-selective LNPs-based mRNA vaccine with minimal inflammation after optimization of ionizable lipids achieved enhanced toxic cellular responses and cancer immunotherapy efficacy in B16F10-OVA subcutaneous tumor and experimental lung metastasis models. Although further work remains, these findings provide a theoretical foundation for the development of potent and low inflammatory mRNA vaccines for cancer immunotherapy, marking a significant leap for mRNA-LNPs technology from targeted delivery to refined immune regulation.

## Supplementary Material

Supplementary figures and tables.

## Abbreviations

LNPs: lipid nanoparticles
mRNA: messenger RNA
APCs: antigen-presenting cells
mRNA-sLNPs: spleen-selective LNPs-based mRNA
pMHC: Peptide-MHC Complex
Tregs: regulatory T cells
MC3: Dlin-MC3-DMA
SA: Stearic acid
IVIS: *in vivo* imaging system
FBS: fetal bovine serum
BMDCs: bone marrow dendritic cells
GM-CSF: granulocyte-macrophage colony-stimulating factor
MFI: mean fluorescence intensity
CCK-8: Cell Counting Kit-8
PI: propidium iodide
PFA: paraformaldehyde
SEM: standard error of the mean
TEM: transmission electron microscopy
SIINFEKL: H2-Kb-restricted OVA257-264 epitope
H&E staining: histopathological examination
