# Supplementary material for: Engineering a spleen-selective mRNA-LNPs vaccine by decoupling the inflammation from cellular immunity-mediated cancer immunotherapy
Source: Theranostics. 2025 Aug 30;15(18):9643–62. doi: 10.7150/thno.118976 (PMC12486252; doi:10.7150/thno.118976)
Supplement: Supplementary file 1 — Supplementary figures and tables. [file thnov15p9643s1.pdf]

Supplementary Materials for

Engineering a spleen-selective mRNA-LNPs vaccine by decoupling the inflammation from cellular immunity-mediated cancer immunotherapy

Table S1. List of antibodies for flow cytometry in this study.

Target	Colour	Clone	Manufacturer	Lot No.
CD11c	PerCP/Cyanine5.5	N418	Biolegend	117327
H-2K^b	FITC	AF6-88.5	Biolegend	116505
H-2K^b bound to SIINFEKL	PE/Cyanine7	25-D1.16	Biolegend	141608
H-2K^b bound to SIINFEKL	APC	25-D1.16	Biolegend	141605
CD80	PE	16-10A1	Biolegend	104708
CD80	FITC	16-10A1	Biolegend	104705
CD80	APC	16-10A1	Biolegend	104714
CD86	PE	A17199A	Biolegend	159204
CD86	APC	GL-1	Biolegend	105012
I-A/I-E	APC/Cyanine7	M5/114.15.2	Biolegend	107627
CD45	Alexa Fluor® 700	I3/2.3	Biolegend	147716
CD11b	APC/Cyanine7	M1/70	Biolegend	101226
CD45R/B220	PE	RA3-6B2	Biolegend	103208
CD4	FITC	GK1.5	Biolegend	100406
CD4	PE	GK1.5	Biolegend	100408

CD8a	PerCP	53-6.7	Biolegend	100732
CD69	Brilliant Violet 421™	H1.2F3	Biolegend	104545
H-2K^b OVA Tetramer	APC		HELIXGEN	HG08T14028

Table S2. Primers used for qRT-PCR.

Gene	Primer sequence (5'-3')
mIl6	5'- TAGTCCTTCCTACCCCAATTTCC-3'
	5'- TTGGTCCTTAGCCACTCCTTC-3'
mIl1β	5'- TTCAGGCAGGCAGTATCACTC-3'
	5'- GAAGGTCCACGGGAAAGACAC-3'
mGapdh	5'-TGTGTCCGTCGTGGATCTGA-3'
	5'-CCTGCTTCACCACCTTCTTGAT-3'

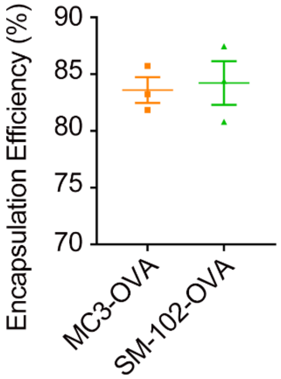

Figure S1. Encapsulation efficiency of MC3 sLNPs-OVA and SM-102 sLNPs-OVA determined by the RiboGreen assay (n = 3 independent samples).

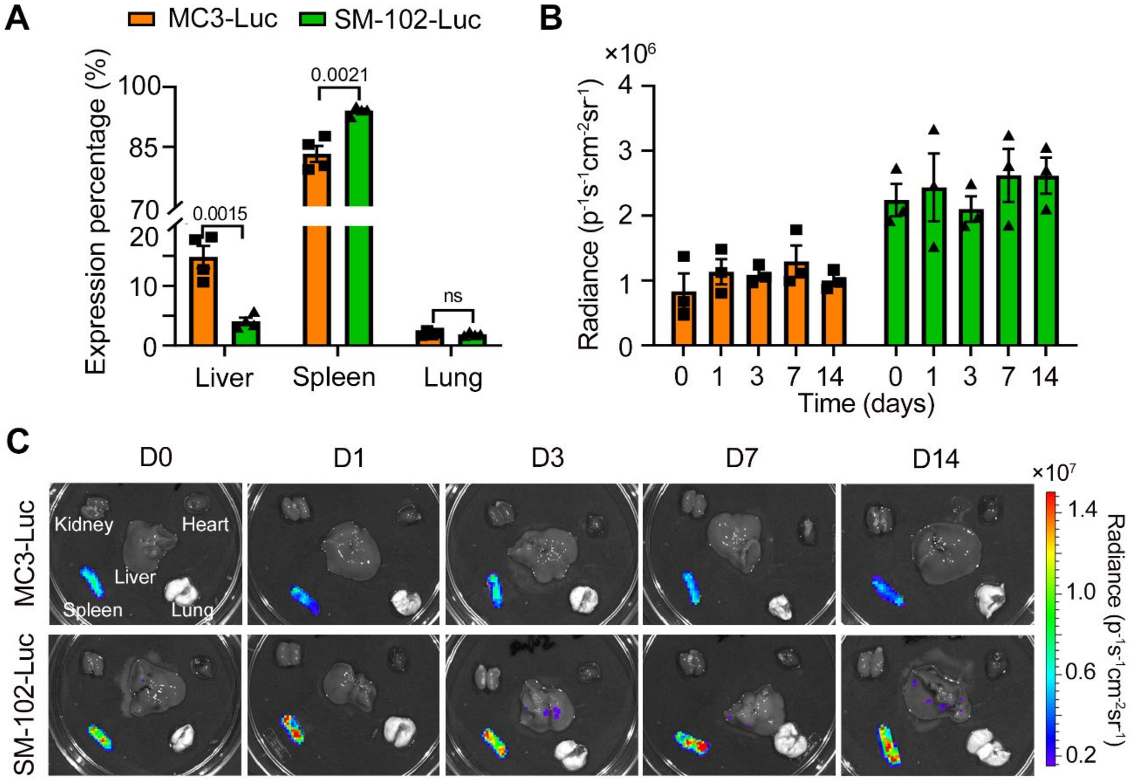

Figure S2. The storage stability of the spleen-targeted formulations was preliminarily investigated by monitoring the translation activity of Luc-mRNA. (A) Expression percentage of Luc-mRNA in the lung, liver, and spleen for MC3 and SM-102 sLNPs 6 hours post i.v. injection on day 0 (n = 4 independent biological samples). (B, C) Quantification of luciferase expression (B) and representative bioluminescence imaging (C)

in the spleen 6 hours post i.v. injection. In vivo Luc expression mediated by spleen-targeted LNPs loaded with Luc-mRNA, constituted freshly or stored at 4 °C for the indicated number of days, followed by equilibration at room temperature for 30 minutes (n = 4 biologically independent samples). Statistical significance was determined by two-way ANOVA with Tukey' s test for (A).

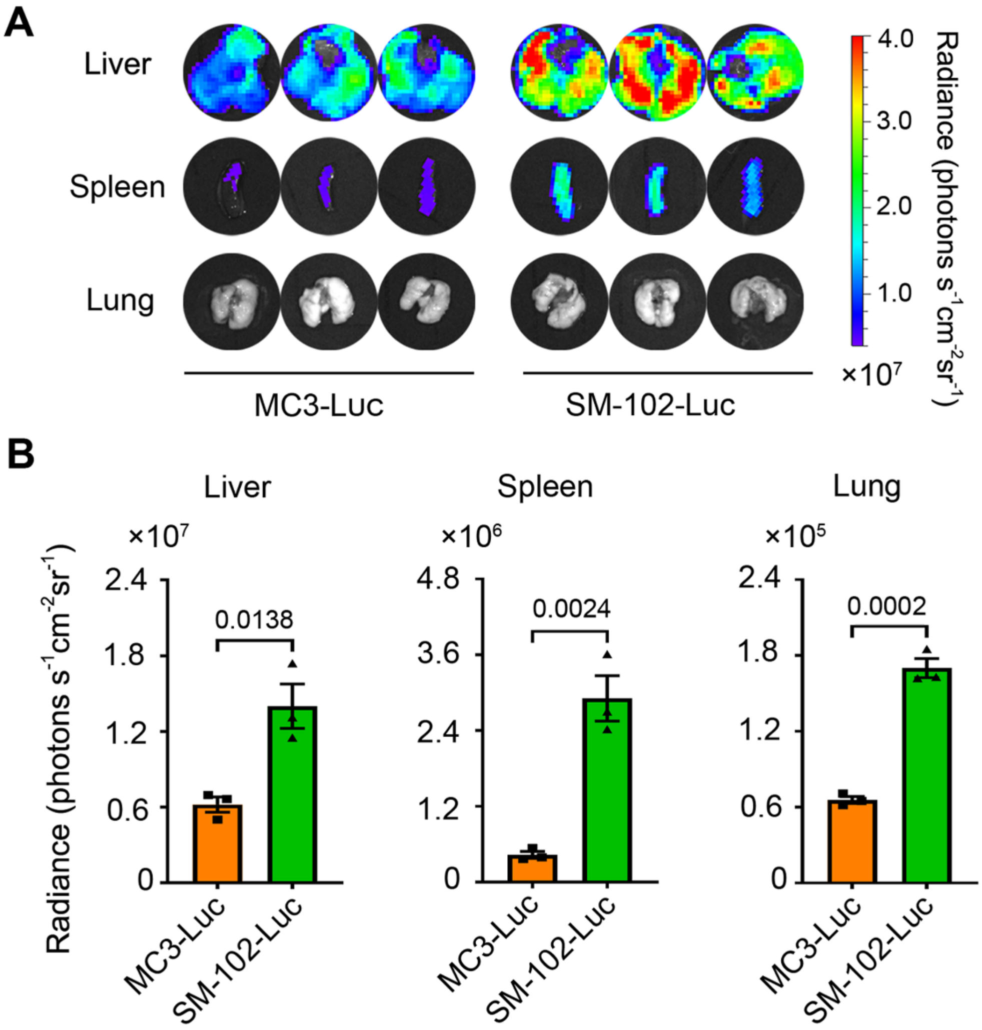

Figure S3. In vivo translation of MC3- and SM-102 LNPs encapsulating Luc-mRNA. (A) In vivo translation of MC3- and SM-102 LNPs-Luc in liver, spleen, and lung as visualized by IVIS imaging. (B) Quantitative analysis of the radiance (p/s/cm²/sr) in the spleen, liver, and lung for MC3- and SM-102 LNPs-Luc (n = 4 independent biological samples). Statistical significance was determined by two-sided unpaired t-test for (B).

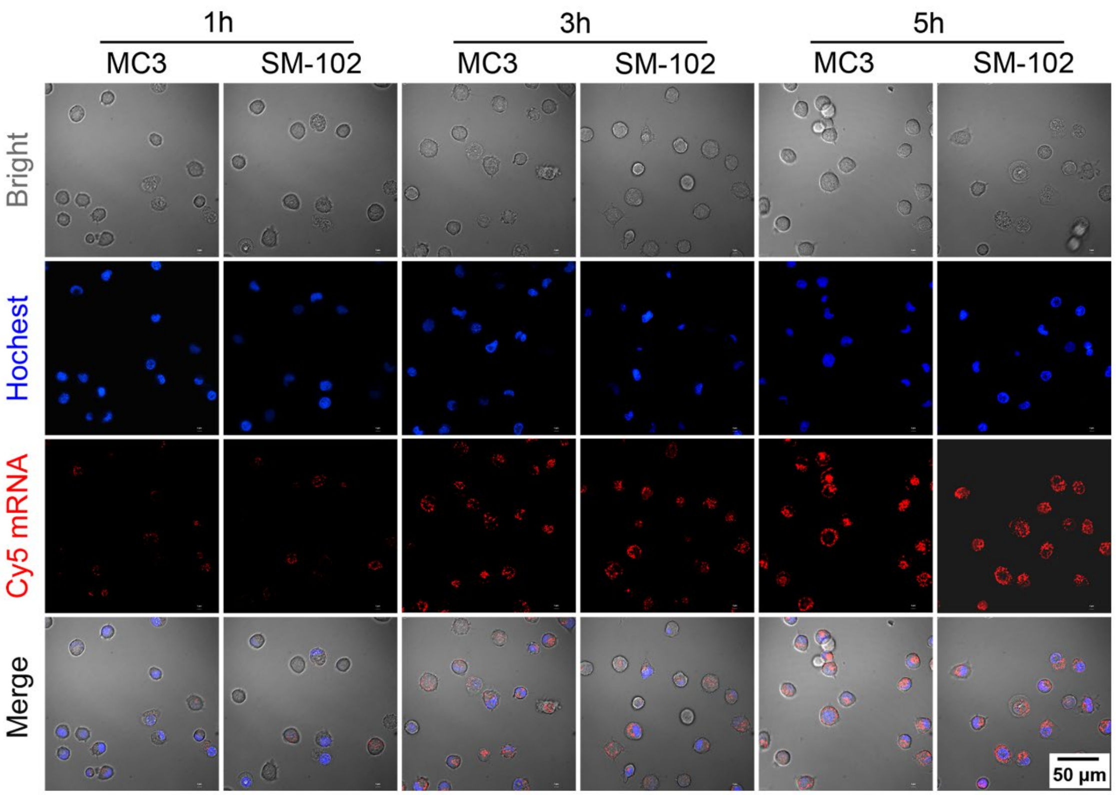

Figure S4. Confocal microscopy images showing the uptake of sLNPs-mRNA by DC2.4 cells at different time points. Cy5-labeled mRNA (red) encapsulated in MC3 or SM-102 sLNPs was incubated with cells for 1 h, 3 h and 5 h. Nuclei were counterstained with Hoechst (blue). Merged images illustrate the intracellular distribution of mRNA relative to nuclear localization. Scale bar: 50 μm.

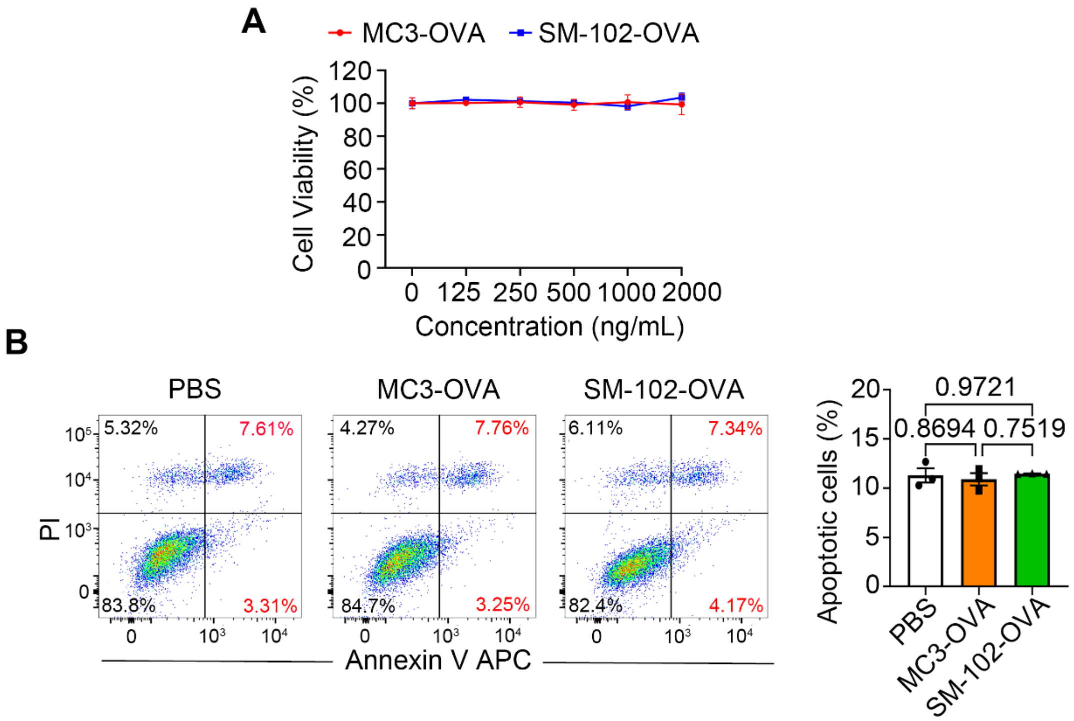

Figure S5. Cytotoxicity evaluation of MC3 and SM-102 sLNPs-OVA. (A) Cell viability of DC2.4 cells treated with MC3 or SM-102 sLNPs-OVA at increasing concentrations (0–2000 ng/mL), measured by CCK-8 assay (n = 3 independent biological samples). (B) Apoptosis analysis of DC2.4 cells treated with PBS (control), MC3 or SM-102 sLNPs-OVA (n = 3 independent biological samples). Statistical significance was determined by one-way ANOVA with Tukey' s multiple comparisons test for (B).

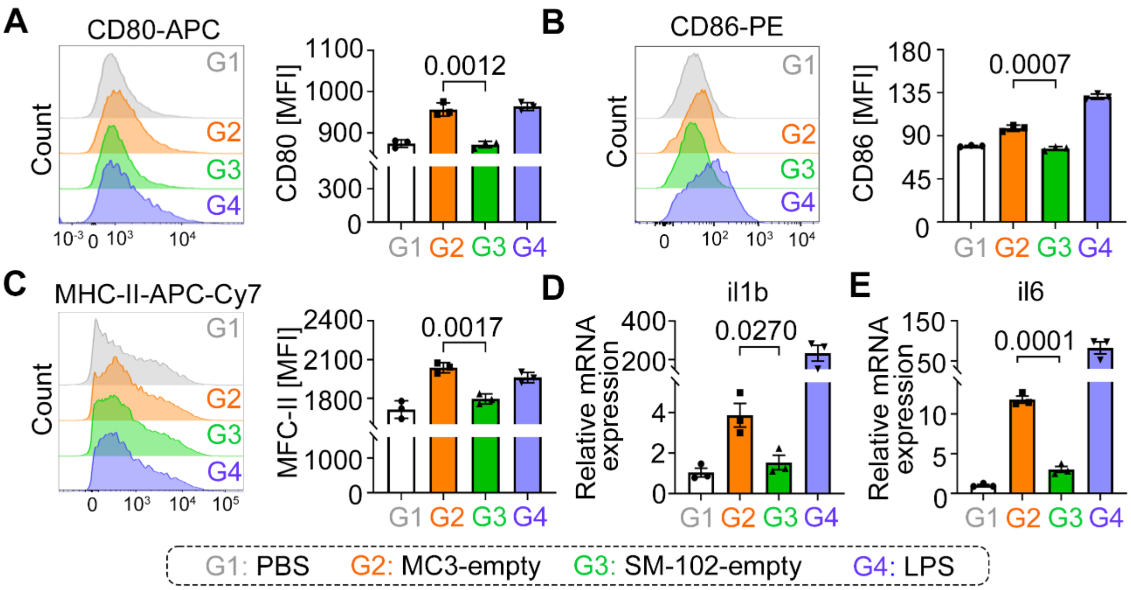

Figure S6. Evaluation of empty MC3 and SM-102 sLNPs on BMDCs activation and cytokine transcription. (A-C) Flow cytometry analysis of surface activation markers (CD80, CD86, MHC-II) on BMDCs after stimulation with empty MC3 or SM-102 sLNPs. (D, E) Quantitative PCR analysis of IL-1β and IL-6 mRNA levels in BMDCs following treatment with empty MC3 or SM-102 sLNPs (n = 3 independent biological samples). Statistical significance was determined by one-way ANOVA with Tukey’ s multiple comparisons test for (A-E).

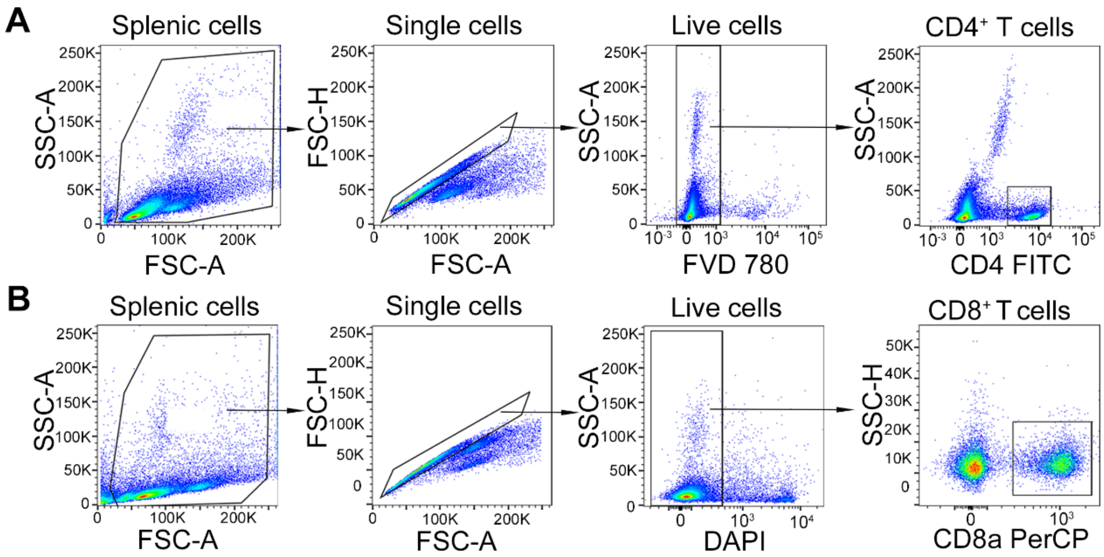

Figure S7. Gating strategy for T cell immune responses in the spleen. (A) Gating strategy for CD4$^+$ T cells in the spleen. (B) Gating strategy for CD8$^+$ T immune responses in the spleen.

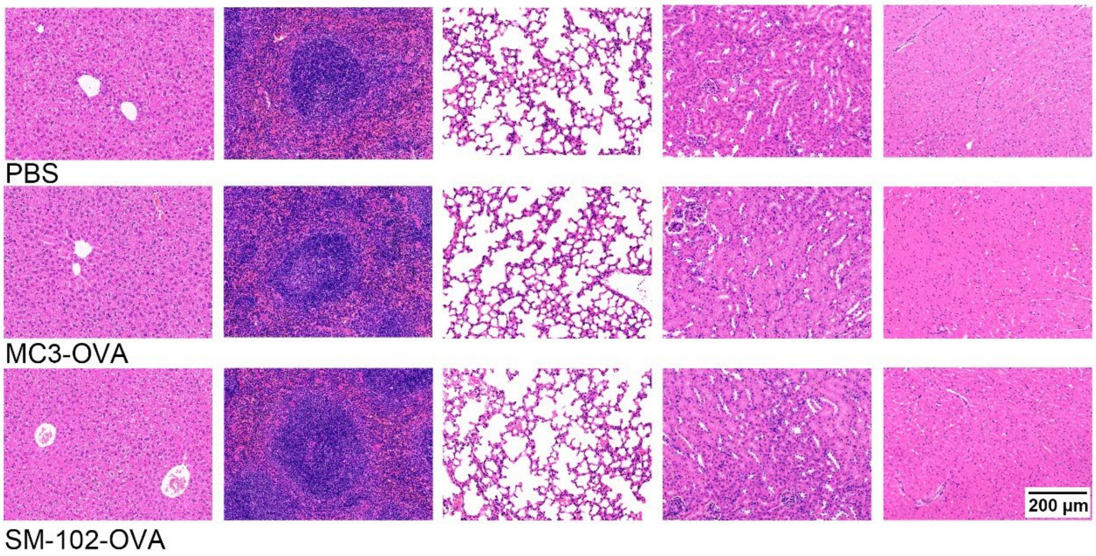

Figure S8. H&E staining was performed on the liver, spleen, lungs, kidney and heart of mice treated with different nanovaccines. Scale bar, 200 μm.

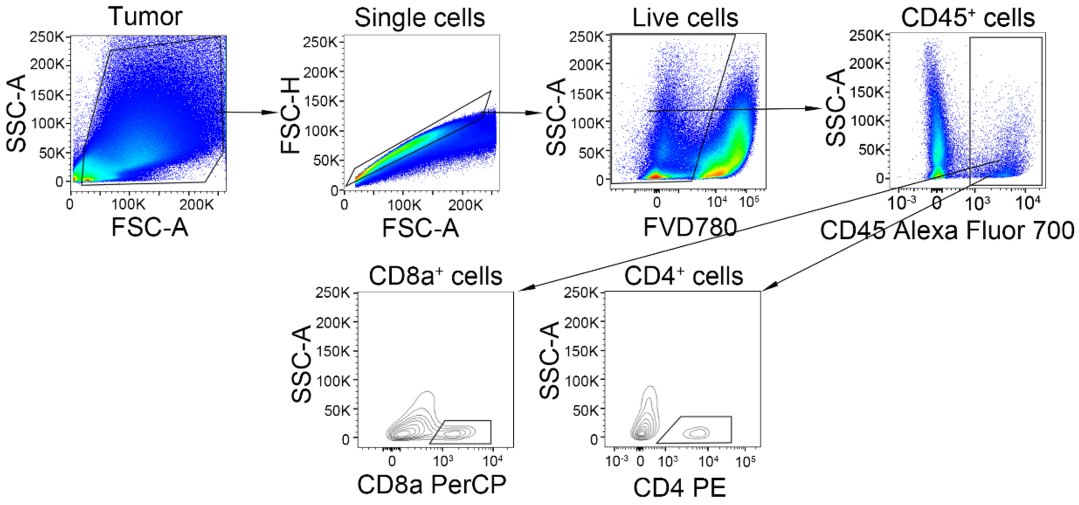

Figure S9. Gating strategy for identifying tumor-infiltrating T cells.

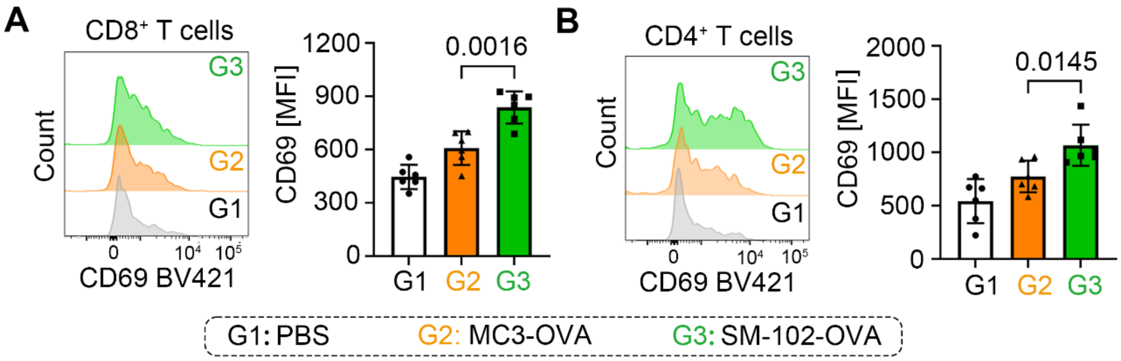

Figure S10. Therapeutic modulation of CD69 expression on tumor-infiltrating T cells. CD69 expression on tumor-infiltrating CD8$^+$ (A) and CD4$^+$ (B) T cells following treatment with PBS, MC3 sLNP-OVA, or SM-102 sLNP-OVA (n = 6 independent biological samples). Statistical significance was determined by one-way ANOVA with Tukey' s multiple comparisons test for (A) and (B).

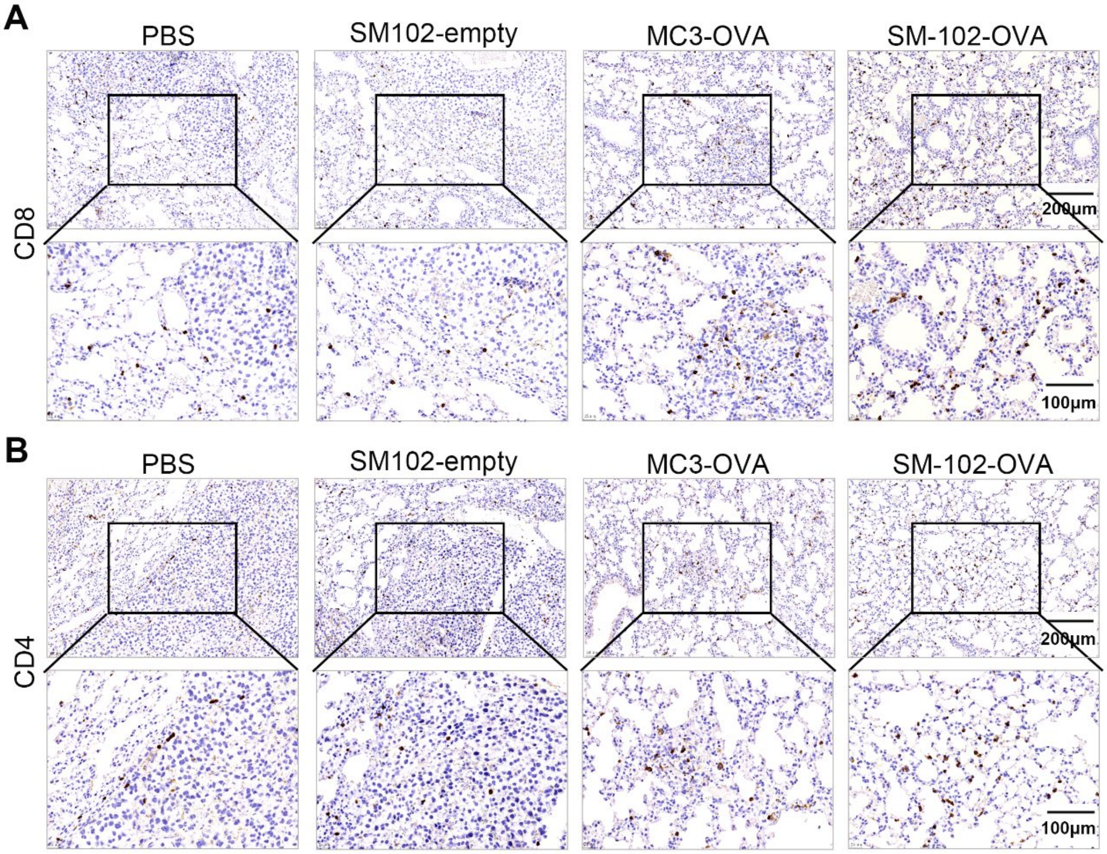

S11. Immunohistochemical analysis of tumor-infiltrating $CD8^+$ and $CD4^+$ T cells. (A, B) Representative images of tumor sections stained for $CD8^+$ T cells (A) and $CD4^+$ T cells (B) in mice treated with PBS (control), MC3 sLNPs-OVA, or SM -102 sLNPs-OVA.
